# miR-33 controls the expression of biliary transporters, and mediates statin- and diet-induced hepatotoxicity

**DOI:** 10.1002/emmm.201201228

**Published:** 2012-07-05

**Authors:** Ryan M Allen, Tyler J Marquart, Carolyn J Albert, Frederick J Suchy, David Q-H Wang, Meenakshisundaram Ananthanarayanan, David A Ford, Ángel Baldán

**Affiliations:** 1 Edward A. Doisy Department of Biochemistry and Molecular Biology, Saint Louis University School of MedicineSaint Louis, MO, USA; 2 The Children's Hospital Research Institute, University of Colorado School of MedicineAurora, CO, USA; 3 Department of Internal Medicine, Saint Louis UniversitySaint Louis, MO, USA; 4 Department of Internal Medicine-Digestive Diseases, Yale University School of MedicineNew Haven, CT, USA; 5 Center for Cardiovascular Research, Saint Louis UniversitySaint Louis, MO, USA

**Keywords:** ABCB11, ATP8B1, cholestasis, miR-33, statins

## Abstract

Bile secretion is essential for whole body sterol homeostasis. Loss-of-function mutations in specific canalicular transporters in the hepatocyte disrupt bile flow and result in cholestasis. We show that two of these transporters, ABCB11 and ATP8B1, are functional targets of miR-33, a micro-RNA that is expressed from within an intron of *SREBP-2*. Consequently, manipulation of miR-33 levels *in vivo* with adenovirus or with antisense oligonucleotides results in changes in bile secretion and bile recovery from the gallbladder. Using radiolabelled cholesterol, we show that systemic silencing of miR-33 leads to increased sterols in bile and enhanced reverse cholesterol transport *in vivo*. Finally, we report that simvastatin causes, in a dose-dependent manner, profound hepatotoxicity and lethality in mice fed a lithogenic diet. These latter results are reminiscent of the recurrent cholestasis found in some patients prescribed statins. Importantly, pretreatment of mice with anti-miR-33 oligonucleotides rescues the hepatotoxic phenotype. Therefore, we conclude that miR-33 mediates some of the undesired, hepatotoxic effects of statins.

→See accompanying article http://dx.doi.org/10.1002/emmm.201201565

## INTRODUCTION

Bile is a complex mixture of bile acids (BAs), cholesterol, phospholipids, proteins and other organic molecules and ions that serves two main purposes: the solubilization of dietary lipids in the intestine, and the removal of waste metabolites through the faeces. Cholestasis refers to a condition with impairment in bile secretion and/or flow, which leads to hepatic injury and, in severe cases, organ failure that requires liver transplantation. The major biliary lipids are secreted across the apical (canalicular) membrane of hepatocytes by three distinct transmembrane transporters: ABCB11 (ATP-binding cassette, sub-family B, member 11; also known as bile salt export pump, BSEP), ABCG5/ABCG8 (ATP-binding cassette, sub-family G, member 5/8; an obligate heterodimer that facilitates cholesterol efflux) and ABCB4 (ATP-binding cassette, sub-family B, member 4; also known as Multi-drug resistance gene MDR3/MDR2 in humans/mice; which pumps phospholipids, mostly phosphatidylcholine, PC) (Esteller, 2008). A fourth transporter, ATP8B1 (ATPase, aminophospholipid transporter, class I, type 8B, member 1), maintains the asymmetry of phospholipid species to promote the required lipid packing of the canalicular membrane for resistance to hydrophobic bile salts and canalicular membrane transport (Paulusma et al, 2006, 2008; Ujhazy et al, 2001). Inactivating mutations in ATP8B1, ABCB11 or ABCB4 result in progressive familial intrahepatic cholestasis (PFIC) type 1, 2 or 3, respectively (Hori et al, 2010; Morotti et al, 2011). Accordingly, these three genes are also known as *FIC-1*, *-2* and *-3*, respectively. Patients with benign recurrent intrahepatic cholestasis (BRIC) also have mutations in any of the latter genes, but the residual activity of the mutant transporter prevents the full PFIC phenotype (Hori et al, 2010; Morotti et al, 2011). Loss-of-function mutations in ABCG5 or ABCG8 result in sitosterolemia (Hubacek et al, 2001; Yu et al, 2002), but not in cholestasis. Mice deficient in any of these transporters phenocopy the human syndromes (Lammert et al, 2004; Pawlikowska et al, 2004; Shah et al, 2010; Wang et al, 2003; Yu et al, 2002). Nevertheless, PFIC/BRIC are thought to develop from the inability to secrete BAs (ABCB11 defect) or phospholipids (ABCB4 defect), or from the inability to maintain canalicular membrane lipid structure (ATP8B1 defect).

We (Marquart et al, 2010) and others (Gerin et al, 2010; Horie et al, 2010; Najafi-Shoushtari et al, 2010; Rayner et al, 2010) recently showed that the evolutionarily conserved *miR-33* is expressed from within intron 16 of *SREBP-2*, and that this miRNA downregulates the expression of the sterol transporter *ABCA1*. Manipulation of miR-33 levels *in vivo* results in changes in circulating levels of high-density lipoproteins (HDL) (Horie et al, 2010; Marquart et al, 2010; Najafi-Shoushtari et al, 2010; Rayner et al, 2010, 2011a, b). Consequently, silencing miR-33 could be useful as a therapeutic intervention to increase plasma HDL in patients with hypercholesterolemia.

Here we test the proposal that miR-33 also modulates hepatic bile metabolism by decreasing the expression of specific sterol transporters in the canalicular membrane of hepatocytes. Our data show that conserved sequences in the 3′UTR of *ABCB11* and *ATP8B1* are functional miR-33-responsive elements (RE), and that manipulation of miR-33 levels by adenoviral over-expression or with antisense oligonucleotides results in altered biliary output *in vivo*. Hence, miR-33 limits sterol efflux in the hepatocyte through both the basolateral membrane (via ABCA1) and the apical membrane (via ABCB11 and ATP8B1). The physiological importance of the murine miR-33 pathway is also supported in experiments using radiolabelled cholesterol, showing that miR-33 silencing increases the amount of labelled sterols recovered from the gallbladder as well as the overall reverse cholesterol transport. We also report that administration of statins, which induce the expression of miR-33, results in decreased hepatic expression of *Abcb11* and *Atp8b1*, but not other canalicular transporters. Finally, we show that silencing miR-33 rescues the hepatotoxicity and lethality caused by co-administration of simvastatin and a cholate-rich diet. We conclude that pharmacologic manipulation of hepatic miR-33 levels might represent a new approach to manage certain cholestatic syndromes.

## RESULTS

### Systemic silencing of miR-33 in mice increases both bile secretion and the expression of hepatic *Abcb11* and *Atp8b1*

**Figure 1:**
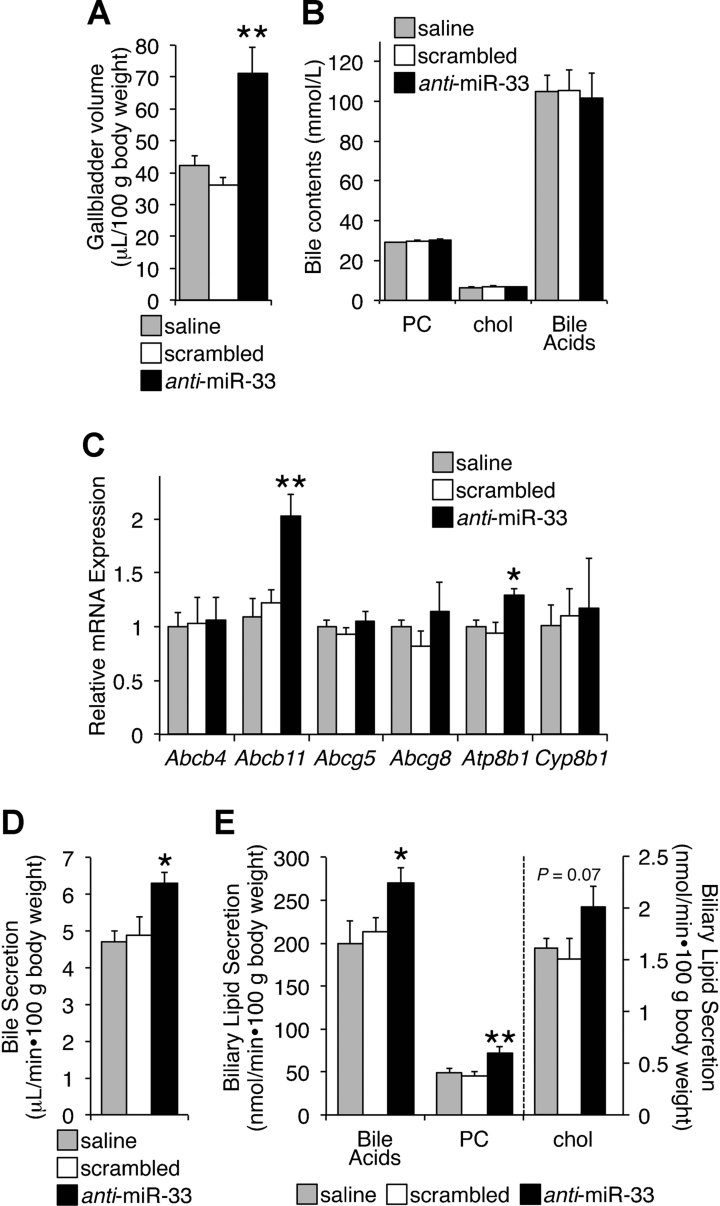
Increased bile secretion following silencing of miR-33 Bile recovered from the gallbladder of mice (*n* = 5) on chow diet, injected with saline, and scrambled or anti-miR-33 oligonucleotides (5 mpk i.v., for 2 consecutive days). Animals were then kept for a week on chow and fasted overnight before sample collection.Levels of phosphatidylcholine (PC), cholesterol (chol) and bile acids present in gallbladder bile in the same mice.Relative expression of hepatic canalicular transporters in the same mice.A different group of mice (*n* = 6–8) was injected as described above. A week later, mice were anesthetized, the bile duct cannulated, and hepatic bile collected for 1 h.Levels of bile acids, phosphatidylcholine (PC), cholesterol (chol) present in bile from the last group of mice. Data shown as mean ± SEM. *p < 0.05; **p < 0.01.

In an effort to understand the *in vivo* physiological importance of miR-33, we injected chow-fed mice with saline, and scrambled or anti-miR-33 oligonucleotides. A week later, we collected different tissue samples. Data show a twofold increase in the volume of bile recovered from the gallbladders of anti-miR-33 animals, compared to controls (Fig 1A). However, the overall concentration of PC, cholesterol and BAs did not change in bile between the three experimental groups (Fig 1B). Next, we tested the expression of several bile-related hepatic canalicular transporters. We found that the messenger RNA (mRNA) levels of both *Abcb11* and *Atp8b1* were significantly increased in the livers of mice injected with anti-miR-33 oligonucleotides, compared to controls (Fig 1C). The mRNA levels of other transporters (*Abcg5*, *Abcg8*, *Abcb4*) and other genes involved in bile homeostasis (*i.e. Shp*, *Cyp7α*) remained unchanged in the same livers (Fig 1C and Supporting Information Fig S1). As expected, previously described miR-33 targets (*Abca1* and *Cpt1α*) were induced by the antisense treatment (Supporting Information Fig S1). To test whether the increase in bile recovery from the gallbladder of mice receiving anti-miR-33 oligonucleotides shown in Fig 1A was due to accelerated bile secretion, an independent group of animals were treated as described above, and after an overnight fasting they were anesthetized and the common bile duct was cannulated to allow the collection of hepatic bile during 60 min. Data in Fig 1D and E show that the secretion rates of total bile, BAs and PC were significantly increased in mice injected with anti-miR-33 oligonucleotides, compared to control animals. The secretion rate of cholesterol also trended up in these same mice, but did not reach statistical significance. The apparently contradictory results shown in Fig 1B and E might be explained by changes in water secretion/reabsorption across the canalicular, ductal and gallbladder epithelium, which are known to modulate bile composition (Portincasa et al, 2008). Nevertheless, the data in Fig 1 demonstrate a functional role for miR-33 on bile secretion.

### *ABCB11* and *ATP8B1* have functional miR-33 responsive sequences in the 3′UTR

**Figure 2:**
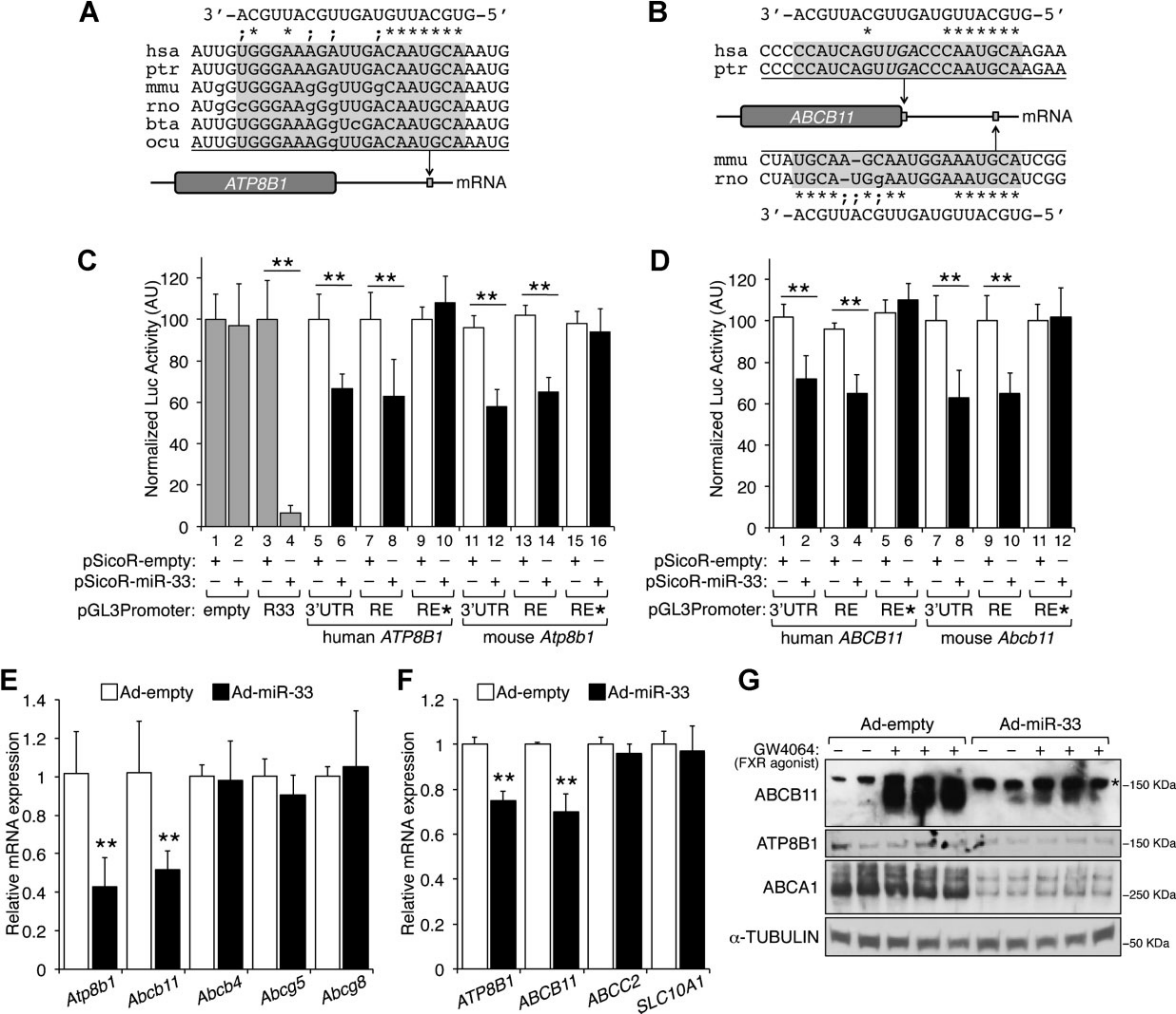
Functional miR-33 responsive elements in the 3′UTR of *ATP8B1* and *ABCB11* **A,B.** Conserved sequences in the 3′UTR of *ATP8B1* and *ABCB11* are partially complementary to miR-33. The element in human *ATP8B1* is located 1877–1897 nt after the stop codon. The element in *ABCB11* overlaps the stop codon in primates, while rodents show a conserved sequence 732–751 nt after the stop codon.**C,D.** Luciferase assays in HEK293 cells using the whole 3′UTR of human or murine *ATP8B1* and *ABCB11*, or a single copy of the responsive elements (RE) identified above, or mutant responsive elements (RE*; where AATGCA was mutated to GGGTTG to prevent complementarity to the seed sequence of the miRNA), co-transfected with (closed bars) or without (open bars) a vector to overexpress miR-33. In grey, data from empty (negative control) and R33 (positive control containing a 100% match to miR-33) reporter vectors.**E,F.** Relative mRNA expression of canalicular transporters in primary murine hepatocytes (*n* = 4 dishes/condition) and human HuH-7 hepatoma cells (*n* = 3 dishes/condition) transduced 48 h with empty or miR-33 adenovirus.**G.** Relative protein levels in HuH7 cells transduced with empty or miR-33 adenovirus. Some cells were incubated for 16 h in the presence of FXR:RXR agonists (2 µmol/L GW4064 : 1 µmol/L 9-*cis*-retinoic acid) to induce ABCB11. Asterisk indicates a non-specific band. Data shown as mean ± SD; **p < 0.01.

Based on the previous results, we hypothesized that both *ABCB11* and *ATP8B1* are direct targets of miR-33. Analysis of the 3′UTR of these genes revealed evolutionarily conserved sequences that are partially complementary to miR-33 (Fig 2A and B). To test whether these sequences are functional, we cloned the 3′UTR of both human and mouse genes, or the putative miR-33 responsive sequences, immediately downstream of a luciferase reporter. Co-transfection with a miR-33-encoding plasmid confirmed that these genes indeed respond to miR-33 (Fig 2C and D). Hence, miR-33 expression resulted in ∼40% decrease in luciferase activity when the reporter is fused to the 3′UTR or the response elements of human/mouse *ATP8B1* (Fig 2C; lanes 5–8 and 11–14) or *ABCB11* (Fig 2D; lanes 1–4 and 7–10). As expected, mutations that prevent the binding of the seed sequence of the miRNA abolished the response to miR-33 (Fig 2C and D; lanes 9–10 and 15–16).

### miR-33 downregulates *ABCB11* and *ATP8B1* in both human and mouse hepatocytes

We next determined whether the mouse and human *ABCB11* and *ATP8B1* genes are regulated following miR-33 overexpression. Hence, mouse primary hepatocytes or human HuH-7 hepatoma cells were transduced with empty or miR-33-encoding adenovirus. Data show that the expression of human/murine *ABCB11* and *ATP8B1* was significantly reduced in cells following overexpression of miR-33 (Fig 2E and F), while mRNA levels of other hepatic transporters (*Abcg5*, *Abcg8*, *Abcb4*, *Abcc2*, *Slc10A1*) remained unchanged (Fig 2E and F). In agreement with the mRNA data, protein levels for both ATP8B1 and FXR-induced ABCB11 were downregulated following miR-33 overexpression (Fig 2G). Collectively, data from Figs 1 and 2 identify *ATP8B1* and *ABCB11* as functional direct targets of miR-33. Additionally, we performed a pulse-chase experiment in HuH7 cells with [^14^C]-cholesterol, to monitor both conversion to [^14^C]-BAs and secretion of labelled sterols. The cells were incubated in media supplemented with 0.2% albumin, but not serum, to minimize loss of the labelled cholesterol via ABCA1 and ABCG1. Data in Supporting Information Fig S2 show that cells overexpressing miR-33 had a significant decrease in their ability to efflux labelled BAs, but not cholesterol, compared to controls. These results suggest that BA secretion is impaired following miR-33 overexpression (consistent with decreased ABCB11 levels), likely resulting in intracellular accumulation of BAs, which suppress further conversion of cholesterol into BAs.

### Hepatic overexpression of miR-33 decreases biliary output in cholate-fed mice

**Figure 3:**
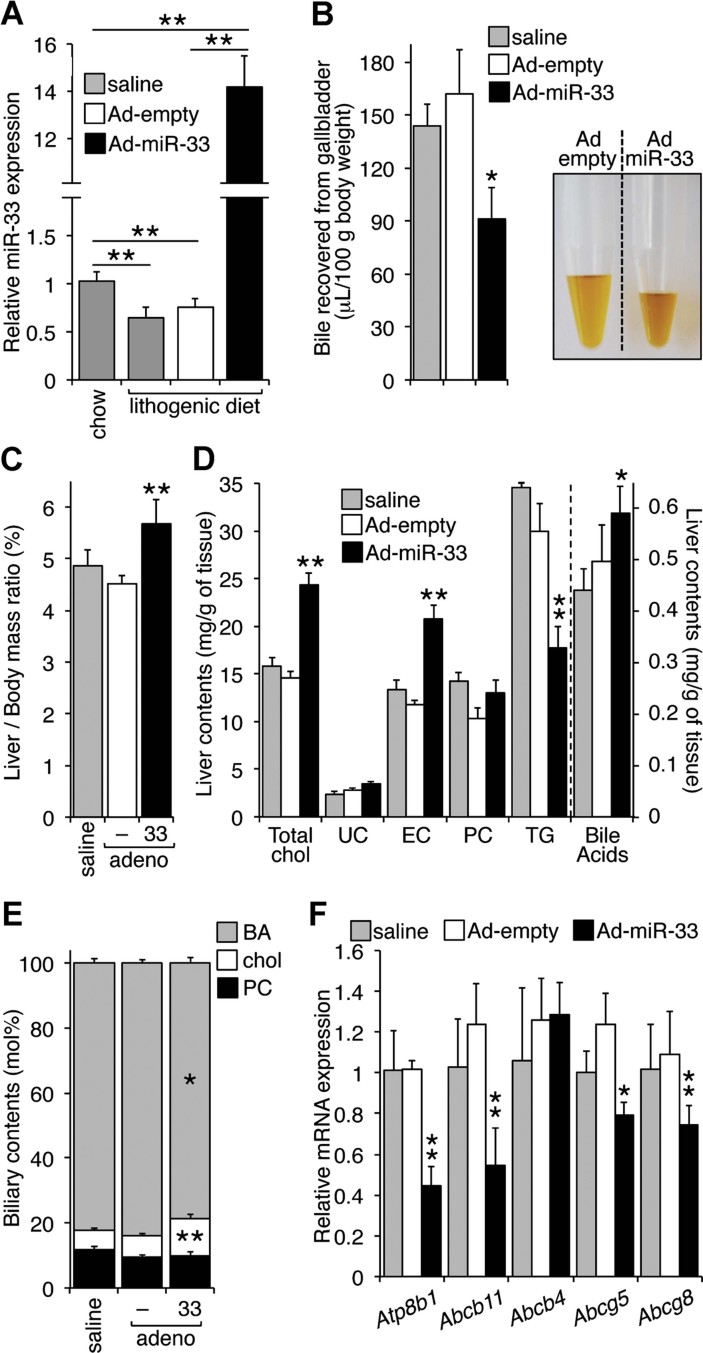
Effect of miR-33 overexpression on diet-induced cholestasis C57BL/6 mice (*n* = 5–7) were kept on chow diet or lithogenic diet for 7 days (lanes 1–2). A different group of animals were transduced i.v. with empty or miR-33 adenovirus (2 × 10^9^ pfu/mouse), and then switched to the lithogenic diet (lanes 3–4). After 7 days, mice were fasted overnight and killed the following morning. Data show relative levels of hepatic miR-33.The volume of bile recovered from the gallbladder is significantly reduced in mice transduced with miR-33. Picture shows pooled bile collected from five mice each in an independent experiment.Liver to body mass ratios.Hepatic levels of bile acids; total, unesterified (UC) and esterified (EC) cholesterol; phosphatidylcholine (PC) and triglycerides (TG).The amounts of bile acids (BA), cholesterol (chol) and phosphatidylcholine (PC) were determined in bile, and expressed as mol% (mol per 100 mol). Compared to mice infused with saline or Adeno-empty, the bile from animals transduced with Adeno-miR-33 showed increased amounts of cholesterol (11.6 ± 1.1 *vs*. 6.6 ± 0.5 *vs*. 5.9 ± 0.6 mol%; miR *v*s. scrambled *vs*. saline; *p* = 0.009), and decreased amounts of bile acids (78.6 ± 1.7 *vs*. 83.7 ± 1.0 *vs*. 82.1 ± 1.4 mol%; miR *v*s. scrambled *vs*. saline; *p* = 0.03), but no change in PC contents (9.8 ± 1.3 *vs*. 9.7 ± 0.6 *vs*. 12.0 ± 0.9 mol%; miR *vs*. scrambled *vs*. saline).Relative expression of hepatic canalicular transporters in the same mice. Data shown as mean ± SD; *p < 0.05; **p < 0.01.

Mice fed a lithogenic diet (21% fat, 1.25% cholesterol and 0.5% cholate) exhibit disrupted bile homeostasis and, after a few weeks, develop cholestasis (Khanuja et al, 1995; Moschetta et al, 2004; Shah et al, 2010; Yu et al, 2005). Our data show that this diet significantly decreases hepatic miR-33 levels after a week, compared to mice fed chow (Fig 3A, lanes 1–2). To test the relevance of hepatic miR-33 under pathological conditions, we performed a diet-induced cholestasis experiment: we injected mice with saline, and empty or miR-33-encoding adenovirus, switched them to the lithogenic diet the following day, and kept them on this diet for 7 additional days. Predictably, the expression of miR-33 was increased in the livers of mice receiving the miR-33 vector (Fig 3A, lanes 3–4). As expected, all the mice fed the lithogenic diet showed an enlarged gallbladder. However, the volume of bile recovered from mice transduced with adeno-miR-33 was ∼45% smaller than that from mice receiving saline or adeno-empty (Fig 3B), suggesting hepatic bile retention. Additionally, the livers from mice receiving miR-33 vectors appeared slightly enlarged, resulting in a significant increase in the liver to body mass ratio (Fig 3C). Analysis of liver contents revealed that overexpression of miR-33 resulted in significant increases in hepatic BAs, total cholesterol and esterified cholesterol (EC), but no changes in unesterified cholesterol (UC) or PC (Fig 3D). For reasons that remain obscure, the amount of triglycerides (TGs) was significantly reduced in the livers of mice transduced with miR-33, compared to control animals (Fig 3D). The molecular basis of this latter effect will require further investigation. Previous reports showed that genes involved in fatty acid β-oxidation and TG metabolism such as CPT1α (carnitine palmitoyltransferase 1α), CROT (carnitine O-octanoyltransferase), HADHB (hydroxyacyl-CoA dehydrogenase/3-ketoacyl-CoA thiolase/enoyl-CoA hydratase, beta subunit), SIRT6 (sirtuin 6) and AMPK1α (AMP-activated protein kinase 1α) are direct targets of miR-33 (Davalos et al, 2011; Gerin et al, 2010). From these studies it was inferred that miR-33 might function to limit fatty acid utilization in hepatocytes and other cell types, and that sustained elevated levels of miR-33 could lead to fatty liver. Based on this literature, we expected to see an increase in hepatic TG contents in mice that overexpressed miR-33, compared to control animals. However, data showing a significant decrease in liver TGs was reproducible in 2 independent experiments (*n* = 5/group). We can only speculate that the impact of miR-33 on lipid metabolism *in vivo* is yet to be fully elucidated, both under normal diet conditions and under dietary challenge (as is the case of data presented in Fig 3). Interestingly, the reduced hepatic expression of *Fasn* in miR-33 overexpressing mice (Supporting Information Fig S3A) could explain the decline in TGs in these mice. However, the reasons for such reduced *Fasn* expression are not clear, since no candidate miR-33 elements are found in human/mouse *FASN*. Additionally, the potential miR-33 RE found in the 3′UTR of human/mouse *SREBP-1* (sterol regulatory element binding protein 1) did not confer response to the miRNA (Supporting Information Fig S3B and C), and the levels of nSREBP-1 were not reduced in the livers of mice overexpressing miR-33 (Supporting Information Fig S3D).

Analysis of the bile revealed a significant decrease in BAs and a modest increase in cholesterol in samples from adeno-miR-33 mice, but no change in PC (Fig 3E). These results are consistent with miR-33 controlling the expression of hepatic transporters involved in sterol mobilization. Indeed, hepatic expression of both *Abcb11* and *Atp8b1* was markedly reduced in mice transduced with adeno-miR-33, compared to adeno-empty (Fig 3F), while the expression of other transcripts did not change between the three groups of mice (Fig 3F and Supporting Information Fig S3A). Importantly, adenoviral-mediated overexpression of miR-33 was also able to reduce the expression of both *Abcb11* and *Atp8b1* in mice fed chow (Supporting Information Fig S3E), suggesting that miR-33 can also modulate basal levels of these transporters *in vivo* under normal diet conditions. Finally, mRNA levels of the sterol transporters *Abcg5* and *Abcg8* were also significantly reduced in the adeno-miR-33 group fed the lithogenic diet (Fig 3F). These latter results were unexpected, since the human and murine *ABCG5* and *ABCG8* genes are not direct targets of miR-33 (Supporting Information Fig S4). Hence, we could not see repression of these latter genes by adenoviral-mediated miR-33 overexpression either in mouse primary hepatocytes (Fig 2E), nor in human HuH7 or HepG2 cells (Supporting Information Fig S4), nor in human Hep3B cells (where exogenous miR-33 also failed to block the induction of *ABCG5* by an LXR agonist (Marquart et al, 2010)). Analysis of the 3′UTR regions of the human and murine *ABCG5/8* genes did not reveal any sequences with perfect complementarity to the seed sequence of miR-33 (Supporting Information Fig S4). However, when cloned downstream of a luciferase reporter, the 3′UTRs of human (but not mouse) ABCG5, and human/mouse ABCG8 conferred a very modest response to miR-33 overexpression (Supporting Information Fig S4). We speculate that these latter results are due to non-conserved, imperfect 6-mer sequences (Supporting Information Fig S4) that bind to miR-33 with low affinity. Hence, the decreased expression of *Abcg5* and *Abcg8* noted in Fig 3F could be the result of off-target effects due to the supra-physiological levels of miR-33 achieved using adenovirus, thus potentially limiting the interpretation of the results. Intriguingly, mice deficient in ABCB11 show decreased hepatic levels of *Abcg5* and *Abcg8* when fed a lithogenic diet (Wang et al, 2003); the molecular mechanism of this cross-talk remains unknown, but authors speculate that it is independent on the levels/activity of LXR (Wang et al, 2003). Whether the drop in *Abcg5/8* levels in the livers in Fig 3F are due to extreme levels of miR-33, or the result of a yet-unknown signalling pathway that links the expression of different bile transporters will require further investigation. Nevertheless, results from Fig 3 and Supporting Information Fig S3 confirm a role for miR-33 on bile homeostasis both under normal chow conditions and following dietary challenge.

### Effect of miR-33 on reverse cholesterol transport (RCT)

**Figure 4:**
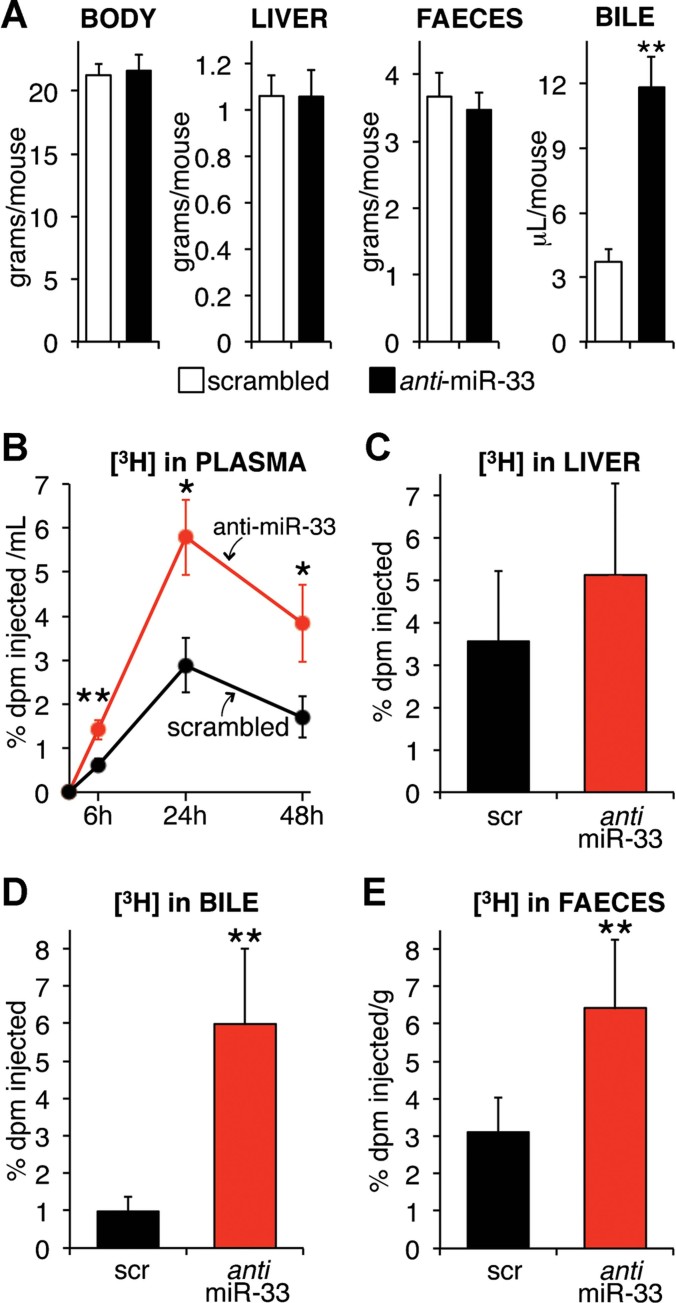
Reverse cholesterol transport is enhanced after systemic miR-33 silencing C57BL/6 mice (*n* = 7) were infused i.v. with 5 mpk scrambled or anti-miR-33 oligonucleotides for 2 consecutive days, and 5 days later received 1 × 10^6^ radiolabelled macrophages by i.p. injection. Mice were kept on chow for 48 h until sacrifice. **A.** Different parameters in mice receiving scrambled (open bars) and anti-miR-33 (closed bars) treatment at the time of sacrifice. Notice the increase in bile recovery from the gallbladder.**B.** Percentage of total injected dpm recovered in the plasma at different time points after injection of radiolabelled cells.**C-E.** Percentage of total injected dpm recovered at the time of sacrifice in liver, bile from the gallbladder and faeces of the same mice. Data shown as mean ± SD; *p < 0.05; **p < 0.01.

RCT mobilizes extrahepatic cholesterol back to the liver for subsequent secretion into bile, and final excretion in the faeces (Khera & Rader, 2010; Rader et al, 2009; Wang et al, 2007; Wang & Rader, 2007). Some authors showed that hepatobiliary secretion is an essential component of the RCT pathway (Nijstad et al, 2011). Others have disagreed (Temel et al, 2010). Remarkably, some patients with cholestasis develop xanthomas that usually dissolve after normal bile homeostasis is restored (Emerick & Whitington, 2002; Englert et al, 2006), suggesting that impairment in bile flow ultimately leads to accumulation of sterols in patients. The previously described target of miR-33, ABCA1, plays an essential role for RCT (Wang et al, 2007). While this manuscript was in preparation, Rayner et al (2011b) showed that systemic silencing of miR-33 promotes RCT, and speculated this effect was the result of increased ABCA1-dependent cholesterol efflux. While it is imaginable that constitutive secretion of bile could funnel an increase of intrahepatic cholesterol derived from ABCA1-induced cholesterol efflux in peripheral tissues, authors have shown that increased hepatic ABCA1 activity promotes the re-secretion of cholesterol into new nascent HDL, thus preventing the mobilization of intrahepatic cholesterol towards the biliary secretory pathway (Annema et al, 2012; Nijstad et al, 2009; Tietge et al, 2008; Wiersma et al, 2009). Consequently, we hypothesized that miR-33-dependent changes in biliary transporters and overall bile secretion (Figs 1–3) contribute, together with ABCA1, to the effect of miR-33 on RCT. To test whether miR-33 expression alters the mobilization of extrahepatic cholesterol into the bile, we injected macrophage foam cells that were radiolabelled with [^3^H]-cholesterol into the peritoneal cavity of animals treated with scrambled or anti-miR-33 oligonucleotides, and followed the fate of labelled sterols for 48 h. Hepatic miR-33 expression was induced 2.1 ± 0.3-fold in mice injected with anti-miR-33 oligos. We did not observe changes in body, liver or faecal mass (Fig 4A). Data show that the amount of labelled cholesterol in plasma, but not in liver, increased in mice receiving anti-miR-33 oligonucleotides, compared to control mice (Fig 4B and C). A large increase in bile volume was noted, again, in the gallbladder of anti-miR-33 mice (Fig 4A). Importantly, and validating our hypothesis, the amount of labelled sterols in the bile was also increased in these latter mice (Fig 4D). Finally, labelled sterols recovered from faeces were increased approximately twofold, compared to control animals (Fig 4D). Subsequent lipid extraction from faeces revealed that the amount of labelled neutral sterols was significantly increased in samples from mice receiving anti-miR-33 treatment, compared to controls (2.31 ± 0.48 *vs*. 0.94 ± 0.18% dpm injected; *p* = 0.025), while the recovery of faecal BAs trended upwards in the same animals, but did not reach statistical significance (3.25 ± 0.47 *vs*. 2.64 ± 0.39% dpm injected; *p* = 0.37). Together, these data are consistent with our hypothesis that miR-33 modulates RCT, likely through the combined regulation of HDL metabolism (via *ABCA1*) and bile metabolism (via *ABCB11* and *ATP8B1*). Collectively, data presented in Figs 1–4 strongly suggest a concerted action of these miR-33 targets. Still, further experiments using mice deficient for each of these transporters will provide definitive answers as to which specific transporter(s) are mediating the effect of anti-miR-33 on RCT.

### Statins repress the expression of miR-33 targets

**Figure 5:**
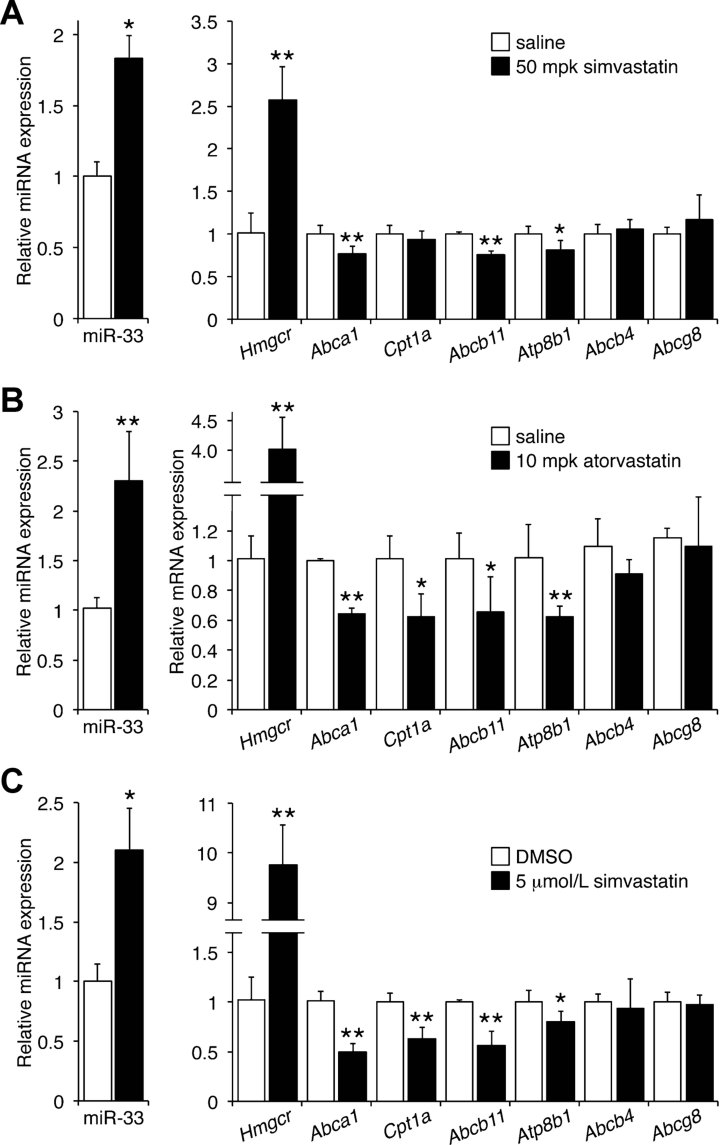
Statins induce miR-33 and reduce the mRNA expression of specific hepatic canalicular transporters **A, B.** C57BL/6 mice (*n* = 5) were gavaged daily with statins, and kept on chow diet. Samples were collected after 7 days, following an overnight fasting.**C.** HuH7 cells were cultured in quadruplicate for 48 h in DMEM supplemented with 2% lipoprotein-deficient serum in the presence or absence of simvastatin. Relative expression of specific genes shown as mean ± SD; *p < 0.05; **p < 0.01.

From a clinical perspective, it may be important that statins not only increase *SREBP-2* expression but also they increase miR-33. Here we show that both simvastatin and atorvastatin induce hepatic miR-33 expression while at the same time decrease the mRNA levels of miR-33 targets *Abca1*, *Cpt1α*, *Abcb11* and *Atp8b1* (Fig 5A and B). Similar results were obtained in HuH7 cells (Fig 5C). These data strongly suggest that patients taking statins might have sustained, decreased levels of hepatic miR-33 targets, including transporters linked to PFIC/BRIC.

### Silencing miR-33 rescues statin- and diet-induced liver damage

**Figure 6:**
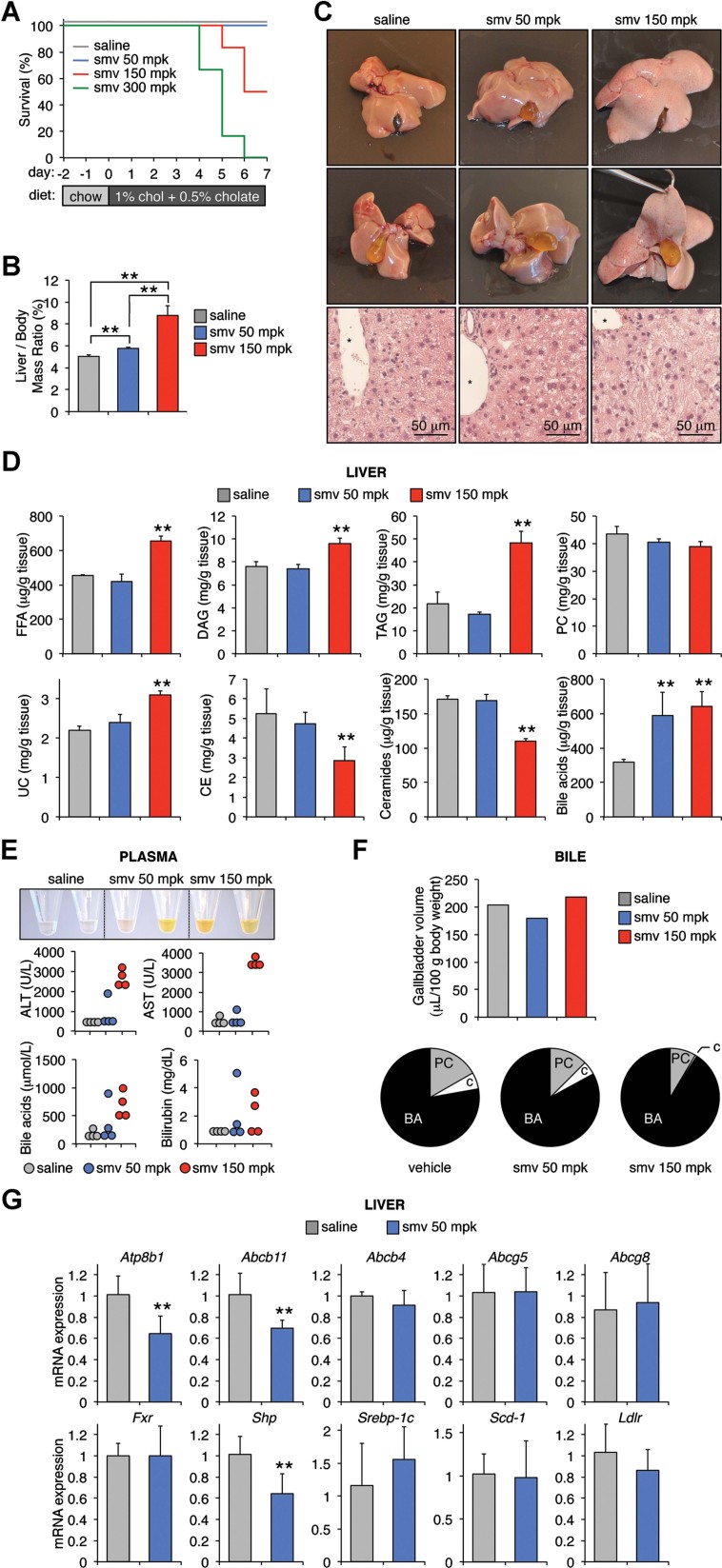
Simvastatin and lithogenic diet induce liver toxicity C57BL/6 mice (*n* = 6) were gavaged daily with simvastatin and fed a lithogenic diet. Samples were collected after 7 days, or when mice appeared moribund. Survival is hampered by simvastatin in a dose-dependent manner.Severe hepatomegaly in mice dosed with 150 mpk simvastatin.Representative macroscopic appearance (upper and middle panels), and haematoxylin and eosin staining of paraffin-embedded sections (lower panels) from the same livers. Note the abnormally swollen cells and pale (*i.e.* steatotic) appearance of the livers in the 150 mpk group.Specific hepatic lipids as determined by ESI-MS, and normalized to tissue weight. FFA, free fatty acids; DAG, diacylglycerides; TAG, triacylglycerides; PC, phosphatidylcholine; UC, unesterified cholesterol; CE, cholesterol esters. **p < 0.01 *versus* saline.Representative samples of plasma, and levels of circulating alanine aminotransferase (ALT), aspartate aminotransferase (AST), bile acids and bilirubin.Bile was recovered from the gallbladder, pooled and the contents of phosphatidylcholine (PC), cholesterol (c) and bile acids (BA) determined with colorimetric kits.Relative expression of hepatic canalicular transporters (upper panel) and other genes involved in bile acid and sterol homeostasis (bottom panel) in samples from mice treated with 0 or 50 mpk simvastatin. Data shown as mean ± SD. **p < 0.01.

We next examined the combined effect of statins and lithogenic diet on mice. In a preliminary experiment, we noted that simvastatin increased miR-33 levels in a dose-dependent manner in chow-fed mice (Supporting Information Fig S5A). Then, we gavaged chow-fed mice the statin (0, 50, 150 or 300 mpk) for 2 days prior to switching them to the lithogenic diet for an additional 7 days. Simvastatin was administered daily during these latter 7 days (Fig 6A), while we monitored body weight and food consumption (Supporting Information Fig S5C). At the end of the experiment, hepatic miR-33 expression was still elevated in the 50 mpk mice, but had decreased in the 150 mpk mice, compared to control animals (Supporting Information Fig S5B). Likely both the statin and the diet contributed to changes in the expression of hepatic miR-33. Importantly, we noted a dose-dependent lethality effect of the statin after mice were switched to the lithogenic diet. Thus, mice on 300 mpk simvastatin had to be euthanized on/before day 5; mice on 150 and 50 mpk simvastatin showed 50 and 100% survival rates, respectively (Fig 6A). This was paralleled by a dramatic dose-dependent increase in liver weight (Fig 6B). The livers of mice receiving 150 mpk simvastatin appeared not only enlarged, but also extremely steatotic (*i.e.* very pale and soft consistency) (Fig 6C); in contrast, livers from mice receiving 50 mpk simvastatin were visually indistinguishable from the livers of control mice (Fig 6C). Electrospray ionization – mass spectrometry (ESI-MS) analysis confirmed the accumulation of lipids, especially TGs, in livers from mice dosed with 150 mpk simvastatin (Fig 6D). Consistent with an intrahepatic cholestatic phenotype, liver BAs levels were also markedly elevated in these latter mice (Fig 6D). Intriguingly, ESI-MS data show a profound remodelling of ceramides in these livers: ceramides containing long-chain fatty acids (16:0, 18:0 and 20:0) were increased, while those containing very long-chain fatty acids (23:0, 24:1 and 24:0) were significantly reduced in the livers of the 150 mpk group (Supporting Information Fig S5D). The (patho)physiological significance of these changes remain obscure. Statin-induced hepatotoxicity was mirrored by increased levels of alanine aminotransferase (ALT), aspartate aminotransferase (AST), bilirubin and BAs in the blood, resulting in bright yellow plasmas (Fig 6E). Although the overall amount of bile recovered from the gallbladders did not differ between groups (Fig 6F), we noted a dose-dependent decrease in PC, small changes in bile salts concentration, and a dramatic decrease in cholesterol in samples from the 150 mpk group (Fig 6F). These changes in bile composition are consistent with abnormal expression/function of canalicular transporters. Finally, we compared the expression of selected transcripts in the livers of mice gavaged saline or 50 mpk simvastatin (mildly cholestatic, based on BA accumulation) (Fig 6G). Data show that both *Abcb11* and *Atp8b1* mRNA were significantly reduced (∼40%) in the simvastatin group; these changes were specific since the expression of other bile-related transporters (*Abcb4*, *Abcg5*, *Abcg8*) did not change (Fig 6G).

**Figure 7:**
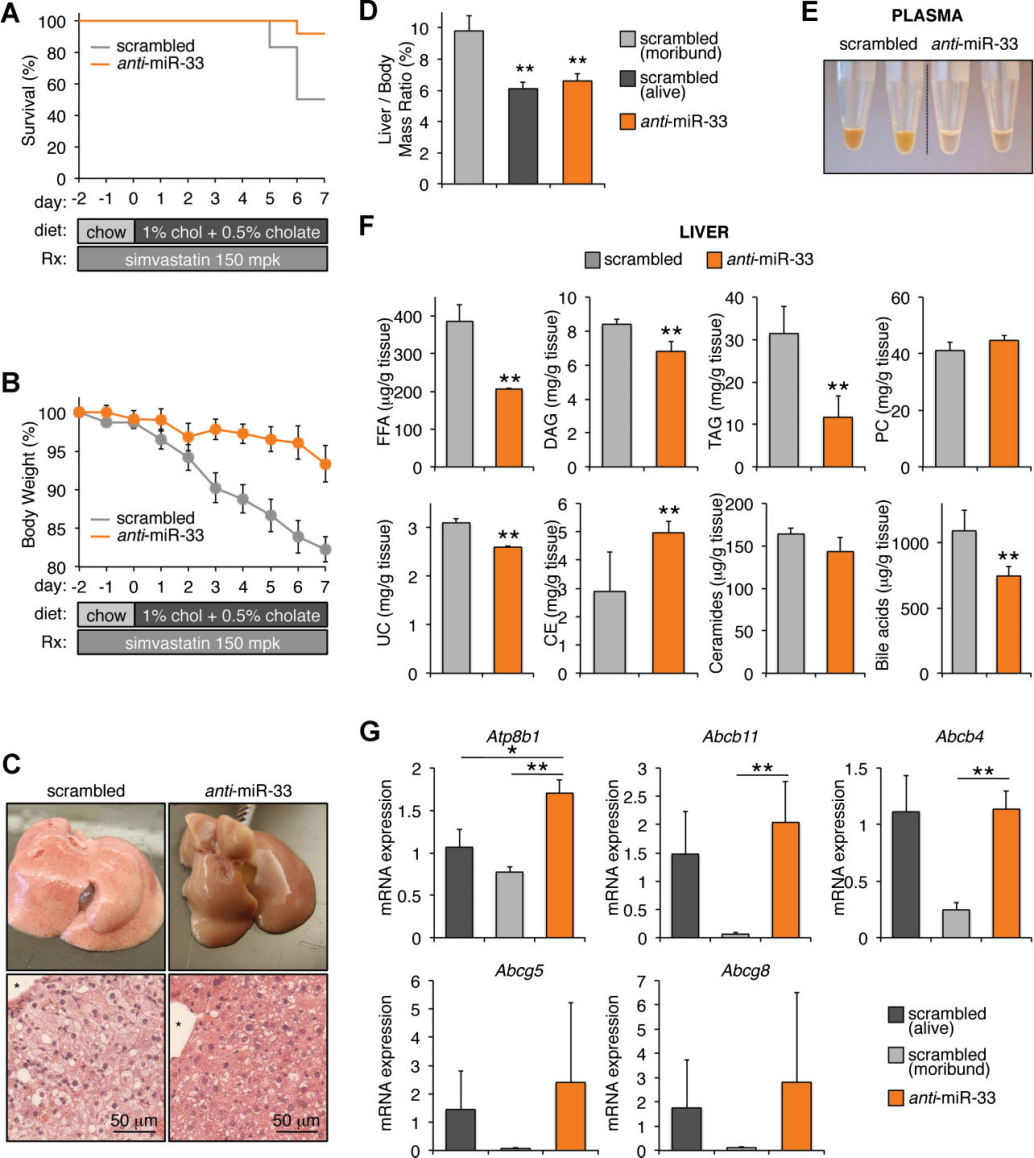
Silencing miR-33 rescues the liver damage induced by simvastatin and lithogenic diet C57BL/6 mice (*n* = 12) were injected i.v. with 5 mpk scrambled or anti-miR-33 oligonucleotides for two consecutive days, and then treated as in Fig 5. Survival curves.Body weight, expressed as % compared to mass on the day of first injection.Representative macroscopic appearance of the livers, and haematoxylin and eosin staining of paraffin-embedded sections.Liver to total body mass ratios. **p < 0.01 *versus* moribund mice.Representative samples of plasma. Note the normalization in colour in the anti-miR-33 group.Amounts of specific hepatic lipids as determined by ESI-MS, normalized to tissue weight (*n* = 4). Acronyms for lipid classes as defined in Fig 5. **p < 0.01 *versus* scrambled.Relative expression of hepatic canalicular transporters (upper panel) and other genes involved in bile acid and sterol homeostasis (bottom panel) (*n* = 5 for scrambled treatment; *n* = 10 for anti-miR-33 treatment). Data shown as mean ± SEM. **p < 0.01 *versus* moribund scrambled mice; *p < 0.05 *versus* surviving scrambled mice.

The results from Fig 6 support a mechanism in which statins induce miR-33, which in turn reduces the levels of both *Abcb11* and *Atp8b1*, resulting in altered bile secretion from hepatocytes, which ultimately leads to liver malfunction under conditions of lithogenic dietary challenge. To validate this model, we next tested whether silencing miR-33 could rescue the phenotype. Hence, mice (*n* = 12) were injected with scrambled or anti-miR-33 oligonucleotides, and then dosed daily with 150 mpk simvastatin and fed the lithogenic diet (Fig 7). Notably, hepatic levels of miR-33 at the end of the experiment were reduced ∼40% in mice receiving the antisense oligonucleotides, compared to control animals (Supporting Information Fig S6A). Data show that, with one exception, all anti-miR-33 mice survived for at least a week, losing ∼5% body weight; in contrast, mice injected with scrambled oligonucleotides showed 50% survival rate and significant body weight loss (Fig 7A and B). Remarkably, the livers from the anti-miR-33 group appeared non-steatotic (Fig 7C), and the liver/body mass ratio was significantly lower than in the scrambled group that succumbed to the treatment (Fig 7D). Additionally, the plasmas from mice receiving anti-miR-33 appeared normal as compared to the yellow-coloured plasma from mice treated with scrambled oligonucleotides (Fig 7E). Rescue of the statin- and diet-induced phenotype was also evident when we analyzed hepatic lipids: anti-miR-33-treated animals showed a significant normalization in lipid contents, compared to control mice (Fig 7F and Supporting Information Fig S6B and C). Finally, we studied the hepatic mRNA levels of selected genes (Fig 7G). Interestingly, the expression of all canalicular transporters, with the exception of *Atp8b1*, was severely decreased in mice that succumbed to the treatment. Additionally, the levels of *Abcg5* and *Abcg8* were, for reasons that are not clear, highly variable within each group of mice (Fig 7G). When only considering those mice that survived 7 days on the diet, treatment with anti-miR-33 oligonucleotides resulted in significant increased expression of *Atp8b1*, but not of *Abcb11* or any other canalicular transporter (Fig 7G). Perhaps the expression of *Abcb11* is already maximal in these livers, due to the diet-induced activation of FXR. In general, the mRNA levels of bile-related genes in surviving mice in the scrambled group were similar to those in the antisense group (Fig 7G), suggesting that the expression of these genes is critical for survival. We also analyzed the mRNA expression of selected hepatic Phase I and II detoxifying genes (Supporting Information Fig S6D). Again, we found large differences in the expression of most of these genes within each experimental group, making the interpretation of the data difficult. We speculate that the increased survival of mice receiving the anti-miR-33 treatment is likely due to complex, coordinated changes in the expression of several genes, which ultimately results in the accelerated clearance of bile and drug and/or diet-derived toxic metabolites. Nevertheless, data in Fig 7 show conclusively that miR-33 mediates statin- and diet-induced hepatotoxicity. Additional experiments using mice deficient in ABCB11 and/or ATP8B1 will provide clues about the relative contribution of each specific transporter to statin-induced, miR-33-dependent hepatotoxicity. The exact hepatoprotective mechanism of anti-miR-33 on statin- and diet-induced toxicity is yet to be determined. We speculate that increased bile flow due to de-repression of miR-33 targets such as *Abcb11* and/or *Atp8b1* contribute to the protective effect, but whether other bile-independent pathways are also necessary for hepatoprotection remains to be established.

## DISCUSSION

**Figure 8:**
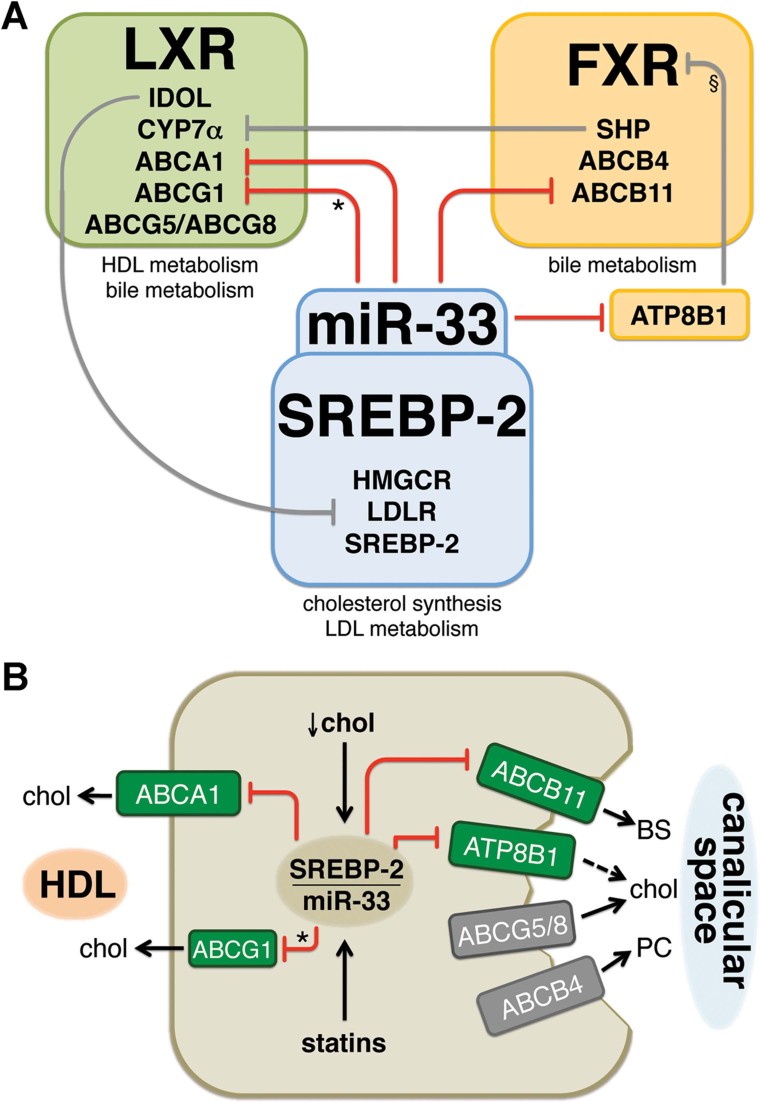
miR-33 limits the mobilization of sterols in hepatocytes through both the sinusoidal and the canalicular membrane miR-33 mediates the cross-talk between the SREBP-2, LXR and FXR pathways by directly modulating the expression of *ATP8B1*, *ABCB11*, *ABCA1* and *ABCG1* (* in mice, but not in humans). Data suggest that miR-33 might also indirectly affect the expression of *ABCG5/8* (see main text for details). ^§^Decreased ATP8B1 activity in human, but not mouse, hepatocytes results in PKC-dependent inactivating phosphorylation of FXR.During episodes of low intracellular cholesterol, or following treatment with statin drugs, miR-33 is transcriptionally induced and reduces the expression of sterol transporters.

Hepatic sterol homeostasis is a complex process that encompasses *de novo* synthesis of cholesterol, secretion and uptake of lipoproteins, conversion of cholesterol to BAs, and bile secretion. Extensive literature shows that three transcription factors (sterol regulatory element binding protein 2, SREBP-2; liver-X-receptor, LXR; and farnesoid X-activated receptor, FXR) orchestrate complementary aspects of sterol metabolism. Our data (Marquart et al, 2010 and this report) and that from other laboratories (Gerin et al, 2010; Horie et al, 2010; Najafi-Shoushtari et al, 2010; Rayner et al, 2010) suggest that miR-33 plays a central role in coordinating those three pathways (Fig 8A). We hypothesize that miR-33 evolved to limit the efflux of hepatocyte sterols, both through the basolateral and apical membranes, in situations of low intracellular cholesterol that induce SREBP-2 (Fig 8B).

Bile acids bind and activate FXR, which in turn promotes increased bile secretion (by inducing *ABCB11* and *ABCB4*) and reduced BAs synthesis (by indirectly repressing *CYP7α*) (Gadaleta et al, 2010; Zhang & Edwards, 2008). Interestingly, FXR targets were found to be reduced in patients with PFIC-1 or following siRNA against *ATP8B1* in humans hepatocytes (Alvarez et al, 2004; Chen et al, 2004; Koh et al, 2009; Martinez-Fernandez et al, 2009), leading to the proposal that FXR activity can be modulated by ATP8B1, perhaps via protein kinase C (Chen et al, 2010; Frankenberg et al, 2008). Other investigators, however, failed to reproduce these observations (Cai et al, 2009). Nevertheless, multiple studies in patients with PFIC/BRIC-1 and -2 support a critical role for both ATP8B1 and ABCB11 on bile secretion (Davit-Spraul et al, 2010; Harris et al, 2005; Kubitz et al, 2005; Morotti et al, 2011; van der Woerd et al, 2010).

Since SREBP-2/miR-33 are transcriptionally induced following treatment with statins (Marquart et al, 2010; Najafi-Shoushtari et al, 2010; Rayner et al, 2010), we hypothesized that miR-33 might account for some of the (side) effects of these drugs. Several reports show that certain patients develop cholestasis following prescription of statins (Batey & Harvey, 2002; de Castro et al, 2006; Merli et al, 2010; Rahier et al, 2008; Ridruejo & Mando, 2002; Torres et al, 2002). While the molecular events in the livers of these patients are unknown, this clinical setting is consistent with our model (Fig 8) in which statin-induced miR-33 represses both ABCB11 and ATP8B1, thus decreasing bile secretion and eventually leading to intrahepatic cholestasis. We recognize, however, that our experimental model is extreme due to the relatively high dose of the statin used and the fact that patients do not normally consume lithogenic diets (*i.e.* a high fat, high cholesterol and high BA diet). It is intriguing to speculate, however, that these patients might carry specific polymorphisms in *ABCB11* or *ATP8B1* that would make them more susceptible to statin-induced miR-33 effects. In general, most physicians are cautious to prescribe statins to patients with underlying liver disease, and the effect of these drugs on patients with primary biliary cirrhosis is controversial (Abu Rajab & Kaplan, 2010; Stanca et al, 2008; Stojakovic et al, 2007, 2010). Nevertheless, our data clearly demonstrate a dose-dependent effect of simvastatin on diet-induced hepatotoxicity and cholestasis in mice. It is important to stress the fact that mice tolerate 300 mpk statins with a concomitant high fat, high cholesterol diet for months (reviewed in Zadelaar et al, 2007), but to our knowledge no previous studies addressed the interaction between statins and cholate feeding. Our data offer conclusive evidence that, at least in mice, statins potentiate diet-induced cholestasis. From a mechanistic perspective, our data show that the phenotype is largely rescued by anti-miR-33 oligonucleotides.

Authors suggested that a therapy that combines statins and anti-miR-33 oligonucleotides may be useful in hyperlipidemic patients by reversing statin-induced, miR-33-mediated repression of ABCA1, which ultimately would increase plasma HDL (Horie et al, 2010; Marquart et al, 2010; Najafi-Shoushtari et al, 2010; Rayner et al, 2010). Our new data show that miR-33 plays a key role in the hepatic response to statins by coordinating the expression of several sterol transporters, and that disruption of the miR-33 pathway prevents statin-induced hepatotoxicity. Data support the hypothesis that miR-33 controls whole-body sterol homeostasis by affecting both HDL biogenesis (via ABCA1), and bile secretion (via ABCB11 and ATP8B1). Primates, but not rodents, express a second miR-33 gene (miR-33b) from an intron of SREBP-1. Importantly, *SREBPs* are differentially regulated by dietary challenges or statin treatment: both transcripts are decreased by fasting and induced by refeeding (but *Srebp-1* is more potently induced than *-2*) (Horton et al, 1998; Bennett et al, 2008), and only *Srebp-2* is induced by statins (Bennett et al, 2008). Additional studies using primates or humanized mice (in which transgenic miR-33b is expressed from an intron of *Srebp-1*) will be necessary to study the impact of SREBP-1-derived miR-33 on bile metabolism. Nevertheless, we speculate that anti-miR-33 oligonucleotides might be useful to manage patients who develop BRIC as a consequence of partial loss of expression and/or function of ABCB11 or ATP8B1.

## MATERIALS AND METHODS

### Materials

Vectors encoding miR-33 were previously described (Marquart et al, 2010). HEK293 and HuH-7 cells (ATCC) were maintained in DMEM supplemented with 10% fetal bovine serum (FBS). Luciferase reporter constructs containing miR-33 response elements or the 3′UTR of human/mouse ATP8B1, ABCB11, ABCG5 and ABCG8 were generated by cloning each sequence into an XbaI site in pGL3-Promoter (Promega). 3′UTR fragments were amplified from human or mouse genomic DNA using Platinum Pfx (Invitrogen). Control (5′-TCCTAGAAAGAGTAGA) and anti-miR-33 (5′-TGCAACTACAATGCA), locked nucleic acid (LNA) oligonucleotides were kindly provided by Miragen Therapeutics Inc. (Boulder, CO). Control oligonucleotides are designed to not target any mouse RNA annotated in NCBI databases. Mice received 5 mg/kg (in 100 µL saline) via tail vein injection for 3 consecutive days, unless otherwise stated.

### Murine studies

C57BL/6 mice were obtained from NCI/Charles River, and maintained in a 12 h/12 h light/dark cycle with unlimited access to food and water. Where indicated, animals (8–10 week old, *n* = 6 per group, unless noted otherwise) were infused via tail vein with adenoviral vectors (2 × 10^9^ pfu) or antisense oligonucleotides (5 mpk, 3 consecutive days). Where indicated, mice were fed a lithogenic diet (Purina 5A8E). Tissues and bile were collected following overnight fasting. RCT experiments were performed as described (Wang et al, 2007). Briefly, mice were injected with oligonucleotides (5 mpk) on days 1, 2, 8 and 9, and on day 15 injected i.p. with 1–1.5 × 10^6^ [^3^H]-cholesterol-loaded bone marrow-derived macrophages. Blood samples were collected 6, 24 and 48 h thereafter. Liver and bile, and the faeces produced during the last 48 h were collected and flash-frozen until used. The amount of radiolabelled sterols was determined by scintillation. Where indicated, mice were dosed daily for 7 days with saline or statin by oral gavage, and killed following an overnight fasting. Bile secretion was measured in anesthetized mice following common bile duct cannulation, which allowed the continued collection of hepatic bile by gravity. Body temperature was maintained during surgery and bile collection at 37 ± 0.5°C. All studies were approved by the IACUC at SLU.

### Primary hepatocytes

Cells were isolated from 8–10-week-old, male C57BL/6 mice fed chow, using Perfusion and Digest buffers (Invitrogen). Cells were resuspended in William's E Medium (Invitrogen) supplemented with Plating Supplements (Invitrogen), plated in 12- or 6-well BioCoat Collagen I plates (BD), and incubated at 37°C and 5% CO_2_ for 6 h. Then, the media was switched to William's E supplemented with Maintenance Supplements (Invitrogen). Where indicated, cells were transduced with Adeno-empty or Adeno-miR-33 adenovirus (MOI 3). Total RNA were extracted 72 h after transduction.

**The paper explained**

PROBLEM:

Patients with hypercholesterolemia are usually prescribed statins. We and others reported on miR-33, an intragenic micro-RNA that is induced by statins and regulates HDL-cholesterol by modulating the expression of the sterol transporter ABCA1 in the liver. Hence, it was proposed that anti-miR-33 oligonucleotides might be used as a new therapeutic approach to manage hypercholesterolemic patients. However, whether miR-33 also controls the expression of other genes important for sterol metabolism is unknown. Here we test the hypothesis that miR-33 modulates the expression of transporters involved in hepatobiliary secretion. Additionally, we study the role of miR-33 on statin-induced liver toxicity.

RESULTS:

MiR-33 extends the function of its host *SREBP-2* by reducing biliary secretion through direct repression of *ABCB11* and *ATP8B1*. We identify conserved sequences in the 3′UTR of these genes responsible for this control, and show that changes in hepatic levels of miR-33 alter both bile secretion rates and bile recovery from the gallbladder. The combination of statins and a lithogenic diet results in a dramatic phenotype (hepatomegaly, liver steatosis, cholestasis and lethality) in mice, that is rescued with anti-miR-33 oligonucleotides. These latter results suggest that miR-33 mediates, at least in part, the hepatotoxic effects of statins.

IMPACT:

Statins induce the expression of miR-33. Patients taking statins likely have sustained, elevated levels of hepatic miR-33. These drugs may decrease the expression of hepatic sterol transporters indirectly, via miR-33. Several clinical reports showed patients who recurrently develop intrahepatic cholestasis when put on statins, but recover after statin withdrawal. Cholestasis results from decreased bile secretion and/or flow. Patients with mutations in *ATP8B1* and *ABCB11* develop PFIC/BRIC-1 and -2, respectively. The data showing the coordinated regulation of sterol transporters by miR-33 and its impact on both HDL and bile metabolism, add to an ever-growing collection of studies showing that miRNAs function as critical fine-tuners in multiple normal and disease-related biological processes. We hypothesize that the increased expression in ATP8B1 and ABCB11 mediated by an anti-miR-33 treatment might functionally overcome the partial loss of activity of these transporters observed in BRIC-1 and BRIC-2 patients, respectively.

### RNA analysis

RNA was isolated from cells or livers with Trizol. Complementary DNAs (cDNAs) were generated from 2 µg of DNase1-treated RNA using Superscript III (Invitrogen) and random hexamers. Real-time PCR was done with Power SybrGreen reagent (Applied Biosystems), using a LightCycler-480 (Roche). Primer sets are available on request. Values were normalized to 36B4 and calculated using the comparative C_t_ method. The expression of miR-33 was normalized to U6, using MiRCURY miRNA assays (Exiqon).

### Protein analysis

Protein extracts were obtained from cells or tissues, as described (Marquart et al, 2010). Fifty micrograms of protein were resolved in 4–12% Bis–Tris gels, transferred to PVDF membranes, and probed with antibodies for ABCB11 (1:500; a gift from Dr. Renxue Wang from the British Columbia Cancer Research Center), ATP8B1 (1:200; SCBT sc-67712), ABCA1 (1:1000; Novus NB400-105), ABCG5 (1:200; Novus NBP1-95209), FXR (1:200; SCBT sc-13063), SREBP1 (1:100; SCBT sc-13551), β-actin (1:5000; SCBT sc-130656) and α-tubulin (1:1000; Sigma T5168), in TBS-Tween20 containing 4% non-fat dry milk. Immune complexes were detected with horseradish peroxidase (HRP)-conjugated secondary antibodies (1:5000; Bio-Rad). Due to lack of specificity we were not able to use these (or different) antibodies to detect ABCB11 and ATP8B1 in liver samples.

### Luciferase reporter assays

Transfection of Hek293 cells was performed in triplicate in 24-well plates by the calcium phosphate method. Luciferase activity was measured 48 h later using the Luciferase Assay System (Promega), and normalized to β-galactosidase activity to correct for small changes in transfection efficiency.

### Plasma analysis

Circulating levels of ALT, AST, bilirubin and total BAs were determined by Advanced Veterinary Laboratory (Saint Louis, MO).

### Lipid analysis

Liver (50 mg) was homogenized in 500 µL of PBS, and lipids were extracted from 100 µL of the homogenate in the presence of internal standards for each lipid class (Bligh & Dyer, 1959). Similarly, bile and plasma were extracted in the presence of internal standards for each lipid class. Lipid species (phospholipids; TGs; cholesterol esters, CE and ceramides) were quantified directly from lipid organic extracts using shotgun lipidomics based on class separation by MS/MS specific methods (Ford et al, 2008; Han & Gross, 2001, 2005). Bile and liver concentrations of cholesterol, bile salts and PC were also determined using enzymatic kits from Wako Chemicals.

## Author contributions

RMA conceived the hypothesis, designed and performed experiments, analyzed the data and wrote the manuscript. TJM designed, performed and analyzed the RCT experiment. CJA, RMA and DAF performed and analyzed the lipidomics studies. MA and FJS performed mRNA experiments in HuH-7 cells. DQ-HW performed common bile duct cannulations for secretion experiments. ÁB conceived the hypothesis, designed experiments and wrote the manuscript.

## Supplementary material

Detailed facts of importance to specialist readers are published as ”Supporting Information”. Such documents are peer-reviewed, but not copy-edited or typeset. They are made available as submitted by the authors.
