# Supplementary material for: miR-33 controls the expression of biliary transporters, and mediates statin- and diet-induced hepatotoxicity
Source: EMBO Mol Med. 2012 Jul 5;4(9):882–95. doi: 10.1002/emmm.201201228 (PMC3491822; doi:10.1002/emmm.201201228)
Supplement: Supplementary file 1 [file emmm0004-0882-SD1.pdf]

EMBO
Molecular Medicine

Manuscript EMM-2012-01228

miR-33 controls the expression of biliary transporters, and mediates statin- and diet-induced hepatotoxicity

Ryan M. Allen, Tyler J. Marquart, Carolyn J. Albert, Frederick J. Suchy, David Q.-H. Wang, Meenakshisundaram Ananthanarayanan, David A. Ford, and Ángel Baldán

Corresponding author: Ángel Baldán, Saint Louis University

Review timeline:

Submission date:	23 January 2012
Editorial Decision:	15 February 2012
Revision received:	24 April 2012
Editorial Decision:	10 May 2012
Revision received:	16 May 2012
Accepted:	23 May 2012

Transaction Report:

(Note: With the exception of the correction of typographical or spelling errors that could be a source of ambiguity, letters and reports are not edited. The original formatting of letters and referee reports may not be reflected in this compilation.)

1st Editorial Decision 15 February 2012

Thank you for the submission of your manuscript to EMBO Molecular Medicine. We have now heard back from the three referees whom we asked to evaluate your manuscript.

Although the referees find the study to be of potential interest, they also raise a number of concerns about the clarity of the text and rationale behind some experiments. Referees #1 and #3 are concerned about the limited mechanistic insights that could be improved and Referee #3 particularly suggests a number of complementary experiments to strengthen the data.

Given the balance of these evaluations, we feel that we can consider a revision of your manuscript if you can address the issues that have been raised within the time constraint outlined below. Please note that it is EMBO Molecular Medicine policy to allow only a single round of revision and that, as acceptance or rejection of the manuscript will depend on another round of review, your responses should be as complete as possible.

Revised manuscripts should be submitted within three months of a request for revision; they will otherwise be treated as new submissions, except under exceptional circumstances in which a short extension is obtained from the editor. Also, the length of the revised manuscript may not exceed 60,000 characters (including spaces) and, including figures, the paper must ultimately fit onto optimally ten pages of the journal. You may consider including any peripheral data (but not methods in their entirety) in the form of Supplementary information.

I look forward to seeing a revised form of your manuscript as soon as possible.

Yours sincerely,

Editor
EMBO Molecular Medicine

***** Reviewer's comments *****

Referee #1:

Review of Allen et al.

In the present paper Allen et al. investigated the role of miR-33 during the regulation of hepatic bile production which is important for whole body sterol homeostasis. miR-33 regulates hepatic bile metabolism by decreasing the expression of specific sterol transporters in the canalicular membrane of hepatocytes. The authors showed that two specific canalicular transporters, ABCB11 and ATP8B1 are functional targets of miR-33, a microRNA that is expressed from within an intron of SREBP-2 (sterol regulatory element-binding protein-2). Manipulation of miR-33 levels by adenoviral over-expression or with antisense oligonucleotides led to changes in biliary output in vivo and bile recovery from the gallbladder. In experiments using radiolabeled cholesterol, they found that systemic miR-33 silencing increased the amount of labeled sterols in the bile recovered from the gallbladder and also the overall reverse cholesterol transport. Administration of statins which induce the expression of miR-33 resulted in decreased hepatic expression of Abcb11 and Atp8b1. Silencing miR-33 in mice rescued the hepatotoxic phenotype and lethality caused by co-administration of simvastatin and a cholate-rich diet. Thus, manipulation of miR-33 levels might be a novel approach to treat several cholestatic syndromes.

The present study is of high interest and clinical relevance but needs some improvement. In particular, the effect of miR-33 on reverse cholesterol transport (RCT) through specific transporters of the HDL or hepatic bile metabolism needs more mechanistic evaluation. I have the following specific concerns and suggestions:

Major points
- In Figure 4B the amount of labeled cholesterol in plasma is increased in mice receiving anti-miR-33 oligonucleotides. However, the amount decreases after 48h. What is the explanation for that observation?
- The authors speculate that miR-33 modulates RCT (reverse cholesterol transport) likely through the combined regulation of HDL metabolism (via ABCA1) and hepatic bile metabolism (via ABCB11 and ATP8B1). The use of siRNAs against the specific transporters (ABCB11 and ATP8B1) during anti-mir33 treatment would indicate which specific transporter mediates the effect of anti-miR-33 on RCT (or whether both equally are involved).
- Protein levels of ABCB11 and ATP8B1 were downregulated following miR-33 overexpression (with or without FXR agonist). What role plays the FXR agonist in your experiment in Fig 2G? This should be also addressed in the MS text.
- What is the reason for altered mRNA levels of bile-related genes in moribund and surviving mice? Anti-33 treated mice have levels closer to moribund than healthy mice.
- Descriptions of the figures (fig legends) need improvement as well as the respective figure allocation. For example, the secretion rates of bile acids, phosphatidylcholine and cholesterol after miR-33 silencing is shown in Fig 1E and not in Fig 1D, as you mentioned. In addition, in the figure legend of Fig 1E is not mentioned at all. The same for Fig S4B. Please correct.

Minor points
- Sometimes one character/letter is missing in a word, for example in the first paragraph of the results: Replace Cyp7_ by Cyp7a; dpm/_L of bile by dpm/ml of bile.
- Some spelling mistakes: Replace leas by leads; replace meterials by materials.
- In Fig 2C and Fig 4A, chart explanation is missing. What do the black, grey and white histograms mean?
- Please add dilution of antibodies used for Western Blot.
- Please add detailed information about the used anti-miR-33 (dose, chemistry, manufaturer ect) as well as about the scrambled controls.

Referee #2 (Comments on Novelty/Model System):

This is an elegant and very well performed study with potential clinical implications. My recommendation is to accept the manuscript after minor revision

Referee #2 (Other Remarks):

In their manuscript entitled "miR-33 controls the expression of biliary transporters, and mediates statin-and diet-induced hepatotoxicity". Allen et al describe an important role for miR-33 in regulating bile secretion through the post-transcriptional regulation of ABCB11 and ATP8B1 transporters. miR-33 is encoded in the Srebp genes and previous studies from this group and others have shown that miR-33 is a critical regulator of cholesterol and fatty acid metabolism. In the present study, Baldan's group identifies 2 new targets that are involved in the bile secretion. Interestingly, anti-miR-33 was able to prevent hepatic toxicity in mice fed with a lithogenic diet and treated with statins, which may have an important therapeutical implication for the recurrent cholestasis found in some patients prescribed with statins. Overall this is an elegant and very well performed study providing new insights into the regulation of lipid metabolism by miR-33. I have some minor concerns:
1. ATPB11 should be ATP8B1 in page 7
2. GW4069 should be GW4064 in Figure 2G
3. The authors should text the labeled-bile acids in cells transfected with miR-33 and incubated with 14C-cholesterol. If miR-33 is targeting ATP8B1 and ABCB11, the accumulation of bile acids will inhibit the FXR-induced bile acid synthesis.
4. It is surprising that miR-33 over-expression reduces TG levels in the liver. The authors should discuss previous publications from other groups where they indentify miR-33 as a key regulator of B-oxidation of fatty acids.
5. The authors conclude that the increase levels of miR-33 in mice treated with statins leads to the hepatoxicity and that anti-miR-33 therapy was able to reverse this effect. This could be different for other miR-33 targets such us ABCA1. The authors showed that ABCA1 levels are reduced in statin-treated mice but several reports have shown that statins are able to increase HDL. Please add some discussion of this phenomenon in the manuscript.
6. The human genome encode for to miR-33 isoforms in the Srebp genes. The manuscript needs more discussion about the potential differences between the effects of anti-miR-33 therapy they found in mice versus humans. How statins regulates SREBP-1 and -2 levels in human liver. Are there some report showing this measurement?. Please discuss this in the manuscript.

Referee #3 (Comments on Novelty/Model System):

Although the majority of the in vivo experiments are well performed, the mouse model used in figures 6-7 is highly artificial and not a model for statin induced cholestasis as indicated by the authors. There is sufficient novel data in figures 1-5 to warrant publication without these additional data, which merely complicate the findings

Referee #3 (Other Remarks):

This manuscript by Allen et al describes how miR-33, a microRNA identified by their group and others as controlling cholesterol and fatty acid metabolism, regulates the expression of biliary transporters and bile secretion. The authors use both knockdown and overexpression techniques to show that miR-33 downregulates the expression of ABCB11 and ATP8B1, and that inhibition of miR-33 leads to increased sterol content of the bile and reverse cholesterol transport. Finally, the authors show that statin-induced cholestasis in mice fed a lithogenic diet, which results in hepatoxicity and lethality, can be rescued by treatment with anti-miR33 oligonucleotides. Although this work is of potential importance to the understanding of miR-33 biology, there are a number of concerns related to the study design and data presented that limit the conclusions that can be drawn, particularly the assertion by the authors that miR-33 mediates some of the undesired hepatotoxic effects of statins.

1. Few details are provided regarding the source and composition of the anti-miR oligonucleotides used in this study. Please provide information regarding the compound, dose, duration, and sequence.
2. The in vivo studies should include a no treatment/vehicle group, as oligonucleotides, even scrambled controls, notoriously have non-specific/off target effects. Thus, in vivo experiments showing effects of miR-33 overexpression or inhibition on ATP8B1/ABCB11 expression, should include both an oligonucleotide control as well as an untreated control group.
3. The data on miR-33 effects on protein expression of its targets is minimal. Given that miRNAs in mammals typically have a more profound effect on protein expression levels, and these are novel miR-33 target genes, Western blots for ABCB11, ATP8B1 (and control genes) for both Fig 1 (anti-miR33) and Fig 3 (Ad-miR33) should be included. Effects of miR-33 on ATP8B1 expression in Fig 2G could be more convincing.
4. In Fig 3, was miR-33 over-expressed only in combination with a lithogenic diet? It would be of interest to see if miR-33 can downregulate basal levels of these transporters under normal diet conditions.
5. Also in Fig 3, the authors do not address why there is such a big difference in miR-33 expression in untreated and Ad-empty treated mice fed a lithogenic diet. Furthermore, as levels of SREBF2, and thus miR-33a, are typically regulated only 2-4 fold in vivo, the 50-fold increase in miR-33 expression in the livers of Ad-miR-33 treated mice (compared to Ad-empty) represents a highly artificial system, which may lead to exaggerated or off target effects such as those on ABCG5 and G8. The authors should be cautioned against overinterpreting such effects.
6. On pg. 9, line 1, the authors note that there is significantly less hepatic triglyceride in mice over-expressing miR-33, though the reasons remain unknown. There is also a decrease in Fasn expression at the mRNA level in these mice. Could this be as a result of a miR-33 target site present in SREBP-1? The authors should examine SREBP-1 protein expression in these livers, particularly given the large error bars in the SREBP-1 mRNA measurement (Fig S3).
7. The authors state that ABCG5 and ABCG8 are not direct targets of miR-33 given that the target sites in the UTR of these genes do not have "perfect complimentarity". However, there are target sites in the UTR of ABCG5 and ABCG8 that appear to be functional, both according to the luciferase data (Fig S3) and some of the mRNA data (Fig 3F). This may be the result of off-target effects due to the high levels of miR-33 overexpression achieved using adenoviruses, and should be carefully investigated. The authors should also include Western blot data for these genes to support their conclusions that ABCG5/8 are not miR-33 targets.
8. The authors switch from using atorvastatin for the in vivo studies in Fig 5, to simvastatin for the remainder of the in vivo work without providing a rationale. This complicates the interpretation of the data.
9. At several points in the manuscript, the authors assert that effects of miR-33 are specific, as mRNA levels of non-miR-33 targets did not change, however the number of genes examined is quite limited. It is not possible to make such assertions unless more extensive gene expression profiling is performed.
10. The rationale for the study design presented in Fig 6 & 7 is weak. First, the authors present miR-33 expression data from vehicle, 50 mpk and150 mpk simvastatin, on both a chow and a lithogenic diet (Fig S4). However, 150mpk does not increase miR-33 on a lithogenic diet-yet this was the dose these authors chose to use in combination with anti-miR33 therapy. On page 14, line 12, the authors state "The results from Fig 6 support a mechanism in which statins induce miR-33, which in turn reduces the level of both ABCB11 and ATP8B1, resulting in altered bile secretion...ultimately leads to liver malfunction". This statement is not supported by the data, where a hepatotoxic dose of simvastatin does not induce miR-33. Moreover, the clinical relevance of these studies is unclear as humans do not consume such a lithogenic diet. Despite the authors' contention that the hepatotoxicity and lethality in mice fed a lithogenic diet concurrent with simvastatin is reminiscent of the recurrent cholestasis found in some patients prescribed statins, this is a highly artificial model that induces tremendous damage to the liver. The mechanisms by which anti-miR-33 exert its positive effects under these conditions are not clear.
11. On p.14 of the results, the authors state that it is unclear whether the decrease in Abcb11 in the livers of statin treated mice is FXR-independent, since the expression of other FXR target genes was either reduced (Shp) or unchanged (Abcb4). By saying this, the authors appear to be challenging their own hypothesis that these changes are occurring through statin-induced upregulation of miR-33. Was FXR protein level measured? The protein levels of the genes shown in figure 6G should be presented.

12. In Fig. 7G, it is unclear how anti-miR-33 treatment causes such a dramatic rescue of Abcb4 levels when this gene is not a target of miR-33. Again, the variability of Abcg5 and G8 measurements are concerning and the mechanism by which anti-miR-33 treatment affects these genes if they are not direct targets is unclear. The figure legend should clearly state the number of mice in each group (moribund, alive and anti-miR-33).

Minor comments:
Fig. 1E is not included in the figure legend

Legend for Figure 4 should be modified to indicate the time point at which samples were taken for c-e

1st Revision - Authors' Response 24 April 2012

Responses to Reviewer 1

Major Point 1. In Figure 4B the amount of labelled cholesterol in plasma is increased in mice receiving anti-miR-33 oligonucleotides. However, the amount decreases after 48h. What is the explanation for that observation?

The increase in circulating labelled cholesterol in mice receiving anti-miR-33 fits with the previously reported role of this miRNA in controlling i) the expression of hepatic ABCA1 and ii) the levels of circulating HDL. The hypothesis (proposed by us and others in the five miR-33 papers in 2010) was that such elevated HDL pool would increase the availability of "acceptors" for extra-hepatic cholesterol. Data provided in Figure 4 in our original manuscript are in agreement with this hypothesis.

The RCT experiments were originally developed by Dan Rader and colleagues at Penn. While certainly tricky, this experimental approach has been used successfully by other investigators in the last few years. However, certain aspects of the approach are highly variable from individual experiment to individual experiment. These latter aspects include not only the % dpm recovered in feces (anywhere from 1% to >5%, depending on the paper), but also the relative amounts of labeled sterols in blood at 24 and 48 h.

We will not bore the reviewer with an extensive list of RCT papers showing a decrease in circulating labeled cholesterol at 24 and 48h. We will just refer to this one: Wang X, Collins HL, Ranalletta M, Fuki IV, Billheimer JT, Rothblat GH, Tall AR, Rader DJ. (2007) Macrophage ABCA1 and ABCG1, but not SR-BI, promote macrophage reverse cholesterol transport in vivo. J Clin Invest 117:2216-2224.
Here the authors study the contributions of those three transporters to RCT, and show six figures that represent six independent RCT experiments. In three of the figures the levels of labeled sterols in blood do not change from 24 to 48 h; in the other three figures, levels drop in a similar way to what we show in Figure 4. The reasons behind these discrepancies are unknown.

In summary, we do not know why the levels of circulating labelled cholesterol decrease from 24 to 48h in our experiments. But nobody knows whether they "should" drop, remain flat, or even increase. From the published data on RCT experiments (>15 papers in PubMed at the time of resubmitting our manuscript), they either drop or remain flat. Since we injected the same cells in both groups of animals, we can only speculate that circulating radiolabeled cholesterol levels are the result of how much labelled cholesterol is "available" to be effluxed from macrophages, and how much HDL is "available" to act as an acceptor for that efflux. Perhaps a decrease in specific activity of cholesterol in the macrophages from 24 to 48 h (due to continuous endogenous synthesis, and loss through efflux) results in the "dilution" of the amount of the labelled sterol available for mobilization towards HDL/circulation. We regret we could not provide the reviewer with a more conclusive answer, but the experimental approach for RCT is limited in this regard.

In any case, the important message from our original Figure 4 is that treatment with anti-miR-33 results in accelerated mobilization of intracellular cholesterol from the macrophages towards the bile and feces.

Major Point 2. The authors speculate that miR-33 modulates RCT (reverse cholesterol transport) likely through the combined regulation of HDL metabolism (via ABCA1) and hepatic bile metabolism (via ABCB11 and ATP8B1). The use of siRNAs against the specific transporters (ABCB11 and ATP8B1) during anti-mir33 treatment would indicate which specific transporter mediates the effect of anti-miR-33 on RCT (or whether both equally are involved).

We fully agree with the reviewer. Indeed, in our original manuscript in two different sections we wrote: "Further experiments using mice deficient for each of these transporters will provide definitive answers as to which specific transporter(s) are mediating the effect of anti-miR-33 on RCT" and "Additional experiments using mice deficient in ABCB11 and/or ATP8B1 will provide clues about the relative contribution of each specific transporter".

We favor doing these experiments in mice deficient on each (or both) canalicular transporters over the use of siRNAs due to the fact that, in general, commercially available siRNA oligos have unknown potency and specificity. In any case, we tried the approach proposed by the reviewer, using pools of 3 different antisense oligos against Abcb11 at different concentrations, but we failed to see reduced expression of ABCB11 mRNA (data not shown) and protein (see Western blot below) in primary hepatocytes. We reasoned that if these oligos could not decrease the expression of the transporter in cells, there was no reason to believe they would work in animals.

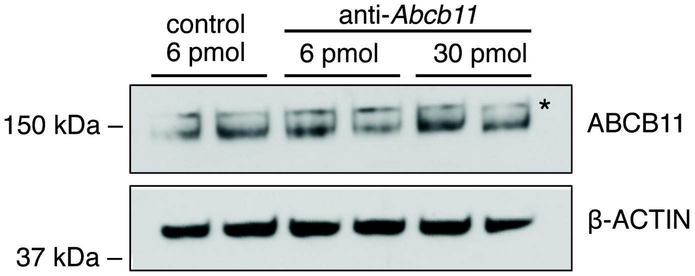

Mouse primary hepatocytes were isolated and plated in 12-well collagen-coated dishes, as described in Methods. Eight h after plating, cells were transfected with 6 pmol or 30 pmol of antisense oligos (Invitrogen Stealth Select RNAi control or MSS219587 anti-Abcb11) using Lipofectamine RNAiMAX, following the manufacturer's recommendations. siRNA against Abcb11 consisted of 3 different oligos, pooled together. Protein extracts were obtained 72 h after transfection, and 50 μg of total protein were analyzed by Western blot, as described in Methods. Asterisk above the 150 KDa marker points to a non-specific band (see Figure 2G in manuscript).

Consequently, at this time we cannot provide a definitive answer as to what is the relative contribution of each of these transporters on the miR-33 effect on RCT. However, we believe the data in our manuscript is compelling to conclude that: i) miR-33 limits the expression of both ABCB11 and ATP8B1, in vitro in both mouse and human hepatocytes and in vivo in mice; ii) decreasing hepatic miR-33 in vivo results in de-repression of both ABCB11 and ATP8B1; iii) a decrease in hepatic miR-33 levels leads to increased bile secretion, while overexpression of hepatic miR-33 results in bile retention; and iv) anti-miR-33 treatment results in accelerated mobilization of cholesterol to the bile and feces.

Interestingly, authors reported that the elevated influx of cholesterol into the liver induced by overexpressing endothelial lipase (1, 2) or sPLA2 (3) did not improve fecal sterol secretion or overall RCT. Some speculate that, under these conditions, ABCA1 can mobilize specific pools of intrahepatic cholesterol for resecretion back into new HDL (4). Indeed, administration of probucol (which inhibits hepatic ABCA1) increases bile secretion and RCT (4, 5). Based on these data, it is unlikely that the effects of anti-miR-33 on RCT can be explained solely by the increase in hepatic ABCA1 expression. Indeed, these latter

studies point to additional transporters, besides ABCA1, that are necessary to partition excess intrahepatic cholesterol towards the bile. We propose that miR-33 coordinates the expression of sterol transporters both at the sinusoidal membrane (ABCA1) and canalicular membrane (ABCB11 and ATP8B1) that are important for the RCT pathway.

We hope that both our efforts with the siRNA oligonucleotides shown above, and this discussion will address the reviewer's concerns.

1. Nijstad N, Wiersma H, Gautier T, van der Giet M, Maugeais C, Tietge UJ (2009) Scavenger receptor BI-mediated selective uptake is required for the remodeling of high density lipoprotein by endothelial lipase. The Journal of biological chemistry 284: 6093-6100

2. Wiersma H, Gatti A, Nijstad N, Kuipers F, Tietge UJ (2009) Hepatic SR-BI, not endothelial lipase, expression determines biliary cholesterol secretion in mice. Journal of lipid research 50: 1571-1580

3. Tietge UJ, Nijstad N, Havinga R, Baller JF, van der Sluijs FH, Bloks VW, Gautier T, Kuipers F (2008) Secretory phospholipase A2 increases SR-BI-mediated selective uptake from HDL but not biliary cholesterol secretion. Journal of lipid research 49: 563-571

4. Annema W, Dikkers A, Freark de Boer J, Gautier T, Rensen PC, Rader DJ, Tietge UJ (2012) ApoE promotes hepatic selective uptake but not RCT due to increased ABCA1-mediated cholesterol efflux to plasma. Journal of lipid research 53: 929-940

5. Yamamoto S, Tanigawa H, Li X, Komaru Y, Billheimer JT, Rader DJ (2011) Pharmacologic suppression of hepatic ATP-binding cassette transporter 1 activity in mice reduces high-density lipoprotein cholesterol levels but promotes reverse cholesterol transport. Circulation 124: 1382-1390

Major Point 3. Protein levels of ABCB11 and ATP8B1 were down regulated following miR-33 overexpression (with or without FXR agonist). What role plays the FXR agonist in your experiment in Fig 2G? This should be also addressed in the MS text.

ABCB11 is a "classic" target of FXR. Protein levels of ABCB11 are very low in untreated cells (either mouse primary hepatocytes or HuH7 cells). The band that appears above 150 KDa in all lanes in the ABCB11 blot is non-specific (labeled with an asterisk). ABCB11 levels are almost undetectable in untreated cells. We used the FXR agonist to induce the expression of ABCB11 (slightly below 150 KDa). Note the substantial induction of ABCB11 in lanes 3-5, compared to lanes 1-2. It is under these conditions that the effect of miR-33 on decreasing the [FXR-induced] expression of ABCB11 becomes more apparent.

Neither ATP8B1 nor ABCA1 are targets of FXR. Accordingly, their levels do not change following treatment with the FXR agonist.

At the reviewer's request, <u>we changed the main text and the figure legend</u> to make it more clear that cells were treated with FXR:RXR agonists to induce the expression of ABCB11. In Figures 2E and 2F we show the decrease in ABCB11 mRNA levels following overexpression of miR-33 in untreated primary hepatocytes and HuH7. Although Abcb11 is expressed at low levels (cells were not incubated with an FXR agonist), the amplification steps during qPCR likely allows us to see the effect of the miRNA. We had also performed additional experiments in cells incubated in the presence or absence of the FXR:RXR ligands and, as expected, miR-33 abrogated the induction of the Abcb11 transcript that

follows FXR activation. We did not include these results in the original manuscript, but the reviewer can see these data below. We do not think this figure adds to the story, but at the reviewer's discretion we will be happy to include it as Supplementary Data.

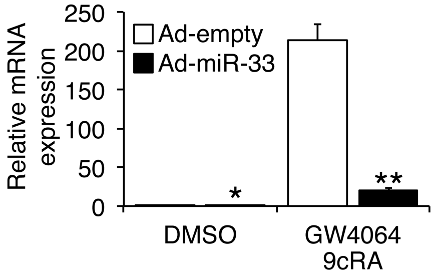

*HuH7 cells (n=3 wells/condition) were transduced with empty- or miR-33-encoding adenovirus, and then incubated for 36 h in the presence of DMSO or FXR:RXR agonists (GW4064:9-cis-retinoc acid; 1 μM each). Total RNA was extracted and the abundance of Abcb11 was determined by real time qPCR, as described in Methods. Data represent mean ± sem; *P ≤ 0.05 miR-33 vs. empty; **P ≤ 0.01 miR-33 vs. empty.*

Major Point 4. What is the reason for altered mRNA levels of bile-related genes in moribund and surviving mice? Anti-33 treated mice have levels closer to moribund than healthy mice.

We regret our choice of colors for that legend. While the legend was strictly correct (darker bars represented surviving mice, and lighter bars represented moribund mice), we placed them in opposite order than the bars in the chart. <u>The figure has been revised, using darker and lighter bars and putting the descriptors in the right order</u>. Consequently, the reviewer will notice that the expression of bile-related genes in anti-miR-33 mice is actually closer to surviving control mice rather than to moribund control mice. Again, we apologize for the confusion in the original figure.

Re the reason behind the altered expression in moribund vs surviving mice, the reviewer is right when s/he points to the fact that the variation in mRNA expression between animals limits the interpretation of data. However, the variability in other parameters measured in these mice (body weight, liver to body mass ratio, hepatic lipid contents) was relatively tight (see rest of Fig 7 and Fig S6). We do not have the answer to why mRNA expression is so variable for bile transporters, but it is certainly puzzling that only mRNA levels showed such variability. We can only speculate, as we did in our original manuscript, that the expression of bile-related genes is particularly critical to support the viability of the mice under conditions of dietary challenge and statin-induced toxicity. That would explain that mice that can maintain the expression of those bile genes above a certain threshold are able to survive the treatment, while those that fail to sustain the expression of bile transporters succumb to the treatment. Expression data in Fig 7G and S6D certainly supports that hypothesis.

<u>The main text now includes the following paragraph:</u> "In general, the mRNA levels of bile-related genes in surviving mice in the scrambled group were similar to those in the antisense group (Fig 7G), suggesting that the expression of these genes is critical for survival. We also analyzed the mRNA expression of selected hepatic Phase I and II detoxifying genes (Fig S6D). Again, we found large differences in the expression of most of these genes within each experimental group, making the interpretation of the data difficult. We speculate that the increased survival of mice receiving the anti-miR-33 treatment is likely due to complex, coordinated changes in the expression of several genes, which ultimately results in the accelerated clearance of bile and drug and/or diet-derived toxic metabolites."

Major Point 5. Descriptions of the figures (fig legends) need improvement as well as the respective figure allocation. For example, the secretion rates of bile acids, phosphatidylcholine and cholesterol after miR-33 silencing is shown in Fig 1E and not in Fig 1D, as you mentioned. In addition, in the figure legend of Fig 1E is not mentioned at all. The same for Fig S4B. Please correct.

We apologize for those mistakes. The text has been corrected.

Minor Point 1. Sometimes one character/letter is missing in a word, for example in the first paragraph of the results: Replace Cyp7_ by Cyp7a; dpm/_L of bile by dpm/ml of bile.

The text has been corrected.

Minor Point 2. Some spelling mistakes: Replace leas by leads; replace meterials by materials.

We apologize for those mistakes. The text has been corrected.

Minor Point 3. In Fig 2C and Fig 4A, chart explanation is missing. What do the black, grey and white histograms mean?

In Figure 2C we used three different colors to provide a visual clue re the different luciferase reporters and/or pSicoR plasmids used in these transfection experiments. As in the rest of the Figure, white bars represent data obtained in experiments where pSicoR-empty is co-transfected, while black bars show data from experiments where pSicoR-miR-33 is co-transfected. Gray bars represent data obtained from negative and positive control luc reporters.

In Figure 4A, white bars represent data from mice injected with scrambled oligos, while black bars show data from mice injected with anti-miR-33 oligos.

The text in both legends has been revised to include this information.

Minor Point 4. Please add dilution of antibodies used for Western Blot.

Primary antibodies were used at the following dilutions: ABCB11 (1:500; a gift from Dr. Renxue Wang from the British Columbia Cancer Research Center), ATP8B1 (1:200; SCBT sc-67712), ABCA1 (1:1,000; Novus NB400-105), ABCG5 (1:200; Novus NBP1-95209), FXR (1:200; SCBT sc-13063), SREBP1 (1:100; SCBT sc-13551), b-ACTIN (1:5,000; SCBT sc-130656) and a-TUBULIN (1,1000; Sigma T5168). Secondary antibodies were used at a 1:5,000 dilution. The text in the manuscript has been modified to include this information.

Minor Point 5. Please add detailed information about the used anti-miR-33 (dose, chemistry, manufacturer etc.) as well as about the scrambled controls.

The following text has been added to the manuscript: "Control (5'-TCCTAGAAAGAGTAGA) and anti-miR-33 (5'-TGCAACTACAATGCA), locked nucleic acid (LNA) oligonucleotides were kindly provided by Miragen Therapeutics Inc. (Boulder, CO). Control oligonucleotides are designed in such a way that they do not target any mouse RNA annotated in NCBI databases. Mice received 5 mg/Kg (in 100 mL saline) via tail vein injection for 3 consecutive days, unless otherwise stated."

Responses to Reviewer 2

Point 1. ATPB11 should be ATP8B1 in page 7

The text has been corrected

Point 2. GW4069 should be GW4064 in Figure 2G

The text has been corrected

Point 3. The authors should text the labelled-bile acids in cells transfected with miR-33 and incubated with 14C-cholesterol. If miR-33 is targeting ATP8B1 and ABCB11, the accumulation of bile acids will inhibit the FXR-induced bile acid synthesis.

As suggested by the reviewer, we transduced human HuH7 cells with empty or miR-33–encoding adenovirus, then pulsed the cells with [14C]-cholesterol for 6 h, and finally chased them for 24 or 48 h in fresh media. We then performed lipid extractions from both the cells and the supernatants as described in Figure 4 to separate labeled neutral sterols (unesterified and esterified cholesterol) and labeled water-soluble sterols (bile acids). These experiments were performed twice, using 8 replicates for each condition. Data in the new Fig S2 show that, the amounts of labeled cholesterol do not change significantly at 24 or 48 h between control and miR-33 overexpressing cells. On the other hand, the secretion of labeled bile acids increases in both groups between 24h and 48 h. But consistent with a role of miR-33 in decreasing bile secretion, the abundance of labeled bile acids in the media is decreased in cells over-expressing miR-33, compared to control cells. Concomitantly, the amount of intracellular labeled bile acids was also decreased in the same cells. Collectively, these results are consistent with a critical role of miR-33 in limiting the ability of hepatocytes to mobilize bile acids towards bile.

We hope our new data will convince the reviewer that miR-33 effectively controls bile acid secretion.

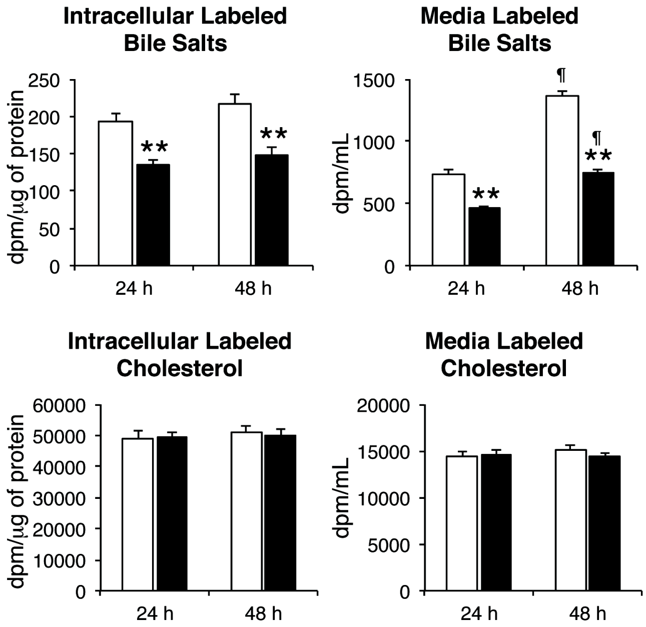

*Conversion of [^{14}C]-cholesterol to [^{14}C]-bile acids is decreased in HuH7 hepatocytes overexpressing miR-33. Cells were seeded in 12-well plates and transduced with empty (white bars) or miR-33-encoding (black bars) adenovirus. After 36 h, cells were pulsed for 6 h with 1μCi/mL [^{14}C]-cholesterol, and then chased in fresh media for 24 or 48 h. Cells and supernatants were collected, and labeled sterols separated as described in Methods for the RCT experiment. Data are shown as mean ± sem; n=8; **P<0.01, miR-33 vs. empty treatment; ¶P<0.01 48 h vs. 24 h.*

Point 4. It is surprising that miR-33 over-expression reduces TG levels in the liver. The authors should discuss previous publications from other groups where they identify miR-33 as a key regulator of B-oxidation of fatty acids.

Reviewers #2 and #3 raised a similar question. At this time the exact mechanism that leads to the decrease in hepatic TG levels following overexpression of miR-33 (as shown in Figure 3) remains to be determined. Gerin et al. (PNAS 2010) and Davalos et al (PNAS

2011) showed that genes involved in fatty acid b-oxidation and triglyceride metabolism (CPT1a, CROT, HADHB, SIRT6 and AMPK1a) are direct targets of miR-33. From these studies it was inferred that miR-33 might function to limit fatty acid utilization in hepatocytes and other cell types, and that sustained elevated levels of miR-33 could lead to fatty liver.

Based on the literature discussed above, we expected to see an increase in hepatic TG contents in mice that overexpressed miR-33, compared to control animals. However, the data was reproducible in 2 independent experiments (n=5/group), and showed a significant decrease in liver TG levels. We can only speculate that the impact of miR-33 on lipid metabolism in vivo is yet to be fully elucidated, both under normal diet conditions and under dietary challenge (as is the case of data presented in Figure 3). Nevertheless, we believe that a lengthy discussion of this topic falls out of the scope of the manuscript and would not add to the story on bile. However, a paragraph has been added on page 9 with the previous discussion.

In response to a follow-up question from reviewer #3, the decrease in Fasn expression in the livers of mice receiving adeno-miR-33 is not due to reduced levels of nSREBP-1 (see data in response to reviewer #3). Additionally, the putative "target site" or a fragment of the 3'UTR in SREBP-1 do not confer response to miR-33 in luciferase assays (see data in response to reviewer #3). Consequently, data do not support the proposal that SREBP-1 is a direct target of miR-33. The reasons behind the decreased expression of Fasn and the reduced hepatic TG levels shown in Fig 3D are not obvious and remain to be established.

Point 5. The authors conclude that the increase levels of miR-33 in mice treated with statins leads to the hepatoxicity and that anti-miR-33 therapy was able to reverse this effect. This could be different for other miR-33 targets such us ABCA1. The authors showed that ABCA1 levels are reduced in statin-treated mice but several reports have shown that statins are able to increase HDL. Please add some discussion of this phenomenon in the manuscript.

Multiple clinical trials have confirmed the role of statins in lowering LDL-cholesterol, thus decreasing the progression of atherosclerosis and the number of cardiovascular events. Some of these studies, such as STELLAR (1), MERCURY I (2), ASTEROID (3) or VOYAGER (4) also reflect a modest, but significant, increase in HDL-cholesterol levels in patients dosed with statins [2–15% across different statins and different doses] (1–4). Intriguingly, while there is a dose-dependent increase in HDL-C with rosuvastatin and simvastatin, higher doses of atorvastatin result in smaller increases in HDL-C. The exact molecular and cellular mechanisms behind this statin-induced increase in HDL-C, and the direct or inverse relationship between doses and HDL-C levels remain obscure, though. Some authors speculate that these effects might be secondary to statin-induced decreased exchange of sterols between HDL and VLDL/LDL, due to reduced plasma CETP levels and/or activity (5, 6). These latter authors speculate that the statin-mediated reduction in pro-atherogenic ApoB-containing lipoproteins might limit the availability for such exchanges, thus resulting in increased pools of HDL-C (5, 6). Consequently, these changes in HDL-C might be independent on direct effects of statins on hepatocyte HDL secretion. Whether other factors, such as dietary components or specific polymorphisms, affect this process is unknown.

*In atherosclerosis-prone mouse models, such as ApoE-/-, Ldlr-/-, and ApoE*3-Leiden mice, the effect of statins on HDL-C levels is even more confusing, and multiple studies report increase, decrease, or no change in HDL-C following treatment with the different statins [extensively reviewed in (7)].*

Decreased expression of hepatic and/or intestinal ABCA1 following treatment with statins has been reported previously (8–11). In our initial paper on the SREBP–miR-33–ABCA1 pathway we speculated "...the specific effects of statins on HDL secretion, metabolism and clearance remain yet to be fully elucidated. We propose that the new [statin-induced] miR-33 pathway impacts the initial, ABCA1-dependent steps of ApoAI lipidation and the subsequent secretion of HDL" (8).

Space limitations preclude a lengthy discussion of this very important topic, but in response to the reviewer's concern *we rewrote the last paragraph of the Discussion section:*
"Authors suggested that a therapy that combines statins and anti-miR-33 oligonucleotides may be useful in hyperlipidemic patients by reversing statin-induced, miR-33-mediated repression of ABCA1, which ultimately would increase plasma HDL (Horie et al, 2010; Marquart et al, 2010; Najafi-Shoushtari et al, 2010; Rayner et al, 2010). Our new data show that miR-33 plays a key role in the hepatic response to statins by coordinating the expression of several sterol transporters, and that disruption of the miR-33 pathway prevents statin-induced hepatotoxicity. Data support the hypothesis that miR-33 controls whole-body sterol homeostasis by affecting both HDL biogenesis (via ABCA1), and bile secretion (via ABCB11 and ATP8B1). Primates, but not rodents, express a second miR-33 gene (miR-33b) from an intron of SREBP-1. Importantly, SREBPs are differentially regulated by dietary challenges or statin treatment: both transcripts are decreased by fasting and induced by refeeding (but Srebp-1 is more potently induced than -2) (Horton et al, 1998; Osborne et al, 2008), and only Srebp-2 is induced by statins (Osborne et al, 2008). Additional studies using primates or humanized mice (in which transgenic miR-33b is expressed from an intron of Srebp-1) will be necessary to study the impact of SREBP-1–derived miR-33 on bile metabolism. Nevertheless, we speculate that anti-miR-33 oligonucleotides might be useful to manage patients who develop BRIC as a consequence of partial loss of expression and/or function of ABCB11 or ATP8B1."

1. Jones P, Davidson MH, Stein EA, et al. STELLAR study group. Comparison of the efficacy and safety of rosuvastatin versus atorvastatin, simvastatin and pravastatin across doses (STELLAR Trial). Am J Cardiol. 2003;92:152-160.

2. Schuster H, Barter PJ, Stender S, et al. Effective Reductions In Cholesterol Using Rosuvastatin Therapy I study group. Effect of switching statins on achievement of lipid goals: Measuring Effective Reductions in Cholesterol Using Rosuvastatin Therapy (MERCURY I) study. Am Heart J. 2004;147:705-713.

3. Nissen SE, Nicholls SJ, Sipahi I, et al. ASTEROID Investigators. Effect of very high-intensity statin therapy on regression of coronary atherosclerosis: the ASTEROID trial. JAMA. 2006;295:1556-1565.

4. Barter PJ, Brandrup-Wognsen G, Palmer MK, Nicholls SJ. Effect of statins on HDL-C: a complex process unrelated to changes in LDL-C: analysis of the VOYAGER Database. J Lipid Res. 2010;51:1546-1553.

5. Guerin M, Lassel TS, Le Goff W, Farnier M, Chapman MJ, Action of atorvastatin in combined hyperlipidemia: preferential reduction of cholesteryl ester transfer from HDL to VLDL1 particles. Arterioscler Thromb Vasc Biol 2000;20:189-197

6. Inazu A, Brown ML, Hesler CB, Agellon LB, Koizumi J, Takata K, Maruhama Y, Mabuchi H, Tall AR. Increased high-density lipoprotein levels caused by a common cholesteryl-ester transfer protein gene mutation. N Engl J Med 1990;323:1234-1238.

7. Zadelaar S, Kleemann R, Verschuren L, de Vries-Van der Weij J, van der Hoorn J, Princen HM, Kooistra T. Mouse models for atherosclerosis and pharmaceutical modifiers. Arterioscler Thromb Vasc Biol. 2007;27:1706-21

8. Marquart TJ, Allen RM, Ory DS, Baldan A (2010) miR-33 links SREBP-2 induction to repression of sterol transporters. *Proc Natl Acad Sci U S A* 107: 12228-12232

9. Wong J, Quinn CM, Gelissen IC, Jessup W, Brown AJ (2007) The effect of statins on ABCA1 and ABCG1 expression in human macrophages is influenced by cellular cholesterol levels and extent of differentiation. *Atherosclerosis*

10. Zeng L, Liao H, Liu Y, Lee TS, Zhu M, Wang X, Stemerman MB, Zhu Y, Shyy JY (2004) Sterol-responsive element-binding protein (SREBP) 2 down-regulates ATP-binding cassette transporter A1 in vascular endothelial cells: a novel role of SREBP in regulating cholesterol metabolism. *J Biol Chem* 279: 48801-48807

11. Genvigir FD, Rodrigues AC, Cerda A, Hirata MH, Curi R, Hirata RD (2011) ABCA1 and ABCG1 expressions are regulated by statins and ezetimibe in Caco-2 cells. *Drug Metabol Drug Interact* 26: 33-36

Point 6. The human genome encodes for two miR-33 isoforms in the Srebp genes. The manuscript needs more discussion about the potential differences between the effects of anti-miR-33 therapy they found in mice versus humans. How statins regulates SREBP-1 and -2 levels in human liver. Are there some report showing this measurement? Please discuss this in the manuscript.

We agree with the reviewer that mice are a somewhat imperfect model to study miR-33 physiology, due to their lack of Srebp-1–derived miR-33b. Multiple laboratories have shown that the expression of SREBP-1 and -2 is regulated differentially not only by nutritional status, but also by statins. We are not aware of any studies that measure the human transcripts in patients following statin treatment, but Tim Osborne and colleagues showed that statins induce mRNA levels of hepatic Srebp-2, but not Srebp-1 (indeed, Srebp-1c levels were significantly decreased following treatment with the drug).

In response to the reviewer's suggestion, we changed the last paragraph of the Discussion to include this topic (see Point 5).

Responses to Reviewer 3

Major Point 1. Few details are provided regarding the source and composition of the anti-miR oligonucleotides used in this study. Please provide information regarding the compound, dose, duration, and sequence.

The following text has been added to the manuscript: Control (5'-TCCTAGAAAGAGTAGA) and anti-miR-33 (5'-TGCAACTACAATGCA), locked nucleic acid (LNA) oligonucleotides were kindly provided by Miragen Therapeutics Inc. (Boulder, CO). Control oligonucleotides are designed in such a way that they do not target any mouse RNA annotated in NCBI databases. Mice received 5 mg/Kg (in 100 mL saline) via tail vein injection for 3 consecutive days, unless otherwise stated.

Major Point 2. The in vivo studies should include a no treatment/vehicle group, as oligonucleotides, even scrambled controls, notoriously have non-specific/off target effects. Thus, in vivo experiments showing effects of miR-33 overexpression or inhibition on ATP8B1/ABCB11 expression, should include both an oligonucleotide control as well as an untreated control group.

Indeed, we included saline-treated mice in the experiments shown in Figs 1 and 3 (oligo-mediated silencing and adeno-mediated overexpression, respectively), but ultimately decided against showing that data in the original manuscript in order to simplify the figures and make them less dense. At the reviewer's request, we now show data from saline-treated mice in those figures (grey columns). The reviewer will notice that the data coming from saline and (scrambled oligo or adeno-GFP) control mice are essentially identical. We hope these new data will easy the reviewer's valid concern.

Major Point 3. The data on miR-33 effects on protein expression of its targets is minimal. Given that miRNAs in mammals typically have a more profound effect on protein expression levels, and these are novel miR-33 target genes, Western blots for ABCB11, ATP8B1 (and control genes) for both Fig 1 (anti-miR33) and Fig 3 (Ad-miR33) should be included. Effects of miR-33 on ATP8B1 expression in Fig 2G could be more convincing.

We agree that it would be desirable to show protein levels. However, as discussed in our original manuscript, we were unsuccessful to produce these data when using several commercially and non-commercially available antibodies against ABCB11 and ATP8B1. It will continue to be a frustrating journey until better tools are available. For example, there

are a few antibodies generated against ATP8B1 that investigators utilized to monitor different mutant variants of this gene found in PFIC/BRIC-1 patients. As expected, these reagents recognize the recombinant protein when overexpressed in heterologous systems (i.e. when plasmids containing those mutant ATP8B1 variants are transfected into Hek293 cells). But when used to interrogate liver extracts (either total extracts or membrane extracts) these antibodies are not specific and/or sensitive enough to detect the native protein.

Fortunately, we are able to use those antibodies to probe both ABCB11 and ATP8B1 protein levels in primary hepatocytes or in cells lines (Fig 2G). We believe that the data presented there are strong, and show a sharp effect of miR-33 on the protein levels of both ATP8B1 and FXR-induced ABCB11.

In general, miRNAs limit the expression of target genes by a combination of mRNA destabilization and translational suppression. Taken together, we believe the data presented in our revised manuscript make a strong case for both ABCB11 and ATP8B1 as direct targets of miR-33. Specifically, we show: i) conserved sequences in the 3'UTR of both genes that are partially complementary to miR-33; ii) that these sequences (or the whole 3'UTR of both the human and murine genes) confers response to miR-33 in luciferase assays, and that mutations in those sequences abolish miR-33 responsiveness; iii) that miR-33 downregulates the mRNA expression of both genes in mouse primary hepatocytes, human HuH7 cells, and mouse livers; iv) that miR-33 decreases the amount of ATP8B1 protein and of FXR-induced ABCB11 protein in HuH7 cells; and iv) that treatment with anti-miR-33 oligonucleotides results in increased hepatic expression of both transporter in vivo. We hope the reviewer concurs with our conclusion, in spite of the lack of protein data from livers.

Major Point 4. In Fig 3, was miR-33 over-expressed only in combination with a lithogenic diet? It would be of interest to see if miR-33 can down regulate basal levels of these transporters under normal diet conditions.

At the reviewer's request, we compared the expression of selected genes in chow-fed mice following transduction with empty- or miR-33-encoding adenovirus. This new experiment is shown below and in the <u>new Fig S3E</u>. Data show that miR-33 overexpression leads to decreased hepatic levels of the sterol transporters Abca1, Abcb11 and Atp8b1, while the relative abundance of other lipid-related genes is unaltered. Consequently, the data suggest that miR-33 can indeed downregulate basal levels of these transporters in vivo under normal diet conditions. <u>Text has been added to the manuscript</u> to reflect these new data: "Importantly, adenoviral-mediated overexpression of miR-33 was also able to reduce the expression of both Abcb11 and Atp8b1 in mice fed chow (Fig S3E), suggesting that that miR-33 can also modulate basal levels of these transporters in vivo under normal diet conditions."

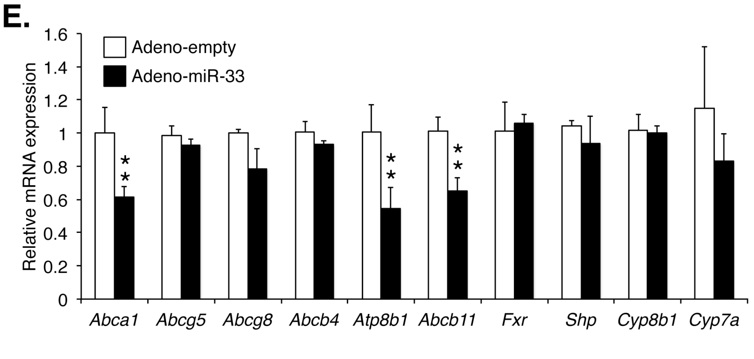

Relative abundance of selected hepatic transcripts in mice transduced with empty or miR-33 adenovirus. Mice were injected as described in Fig 3, and fed a standard chow diet for 7 additional days. The expression of selected genes was measured as described in Methods.

Major Point 5. Also in Fig 3, the authors do not address why there is such a big difference in miR-33 expression in untreated and Ad-empty treated mice fed a lithogenic diet. Furthermore, as levels of SREBF2, and thus miR-33a, are typically regulated only 2-4 fold in vivo, the 50-fold increase in miR-33 expression in the livers of Ad-miR-33 treated mice (compared to Ad-empty) represents a highly artificial system, which may lead to exaggerated or off target effects such as those on ABCG5 and G8. The authors should be cautioned against overinterpreting such effects.

We apologize for the mistake we made in the original in Fig 3A re the expression levels of miR-33. The data in lanes [1-2] and lanes [3-4] were not properly normalized. This figure has been revised and now shows that the expression of hepatic miR-33 does not change significantly between uninjected mice on diet and mice transduced with empty adenovirus on diet (Fig 3A, lanes 2 and 3).

Consequently, the hepatic expression of miR-33 in mice transduced with adenovirus and fed the lithogenic diet is induced ~19-fold, compared to empty-adeno mice on diet, and ~13-fold compared to mice on chow diet (Fig 3A). The reviewer is correct in his/her appreciation that this level of overexpression is quite high and could potentially lead to non-physiological effects. We agree with the reviewer that such exaggerated increase in miR-33 levels might result in off-target effects, such as those in Abcg5 and Abcg8 (see Major Point #7 below for further discussion on this topic). Text has been added to the manuscript stating this potential limitation.

Major Point 6. On pg. 9, line 1, the authors note that there is significantly less hepatic triglyceride in mice over-expressing miR-33, though the reasons remain unknown. There is also a decrease in Fasn expression at the mRNA level in these mice. Could this be as a result of a miR-33 target site present in SREBP-1? The authors should examine SREBP-1 protein expression in these livers, particularly given the large error bars in the SREBP-1 mRNA measurement (Fig S3).

Reviewers #2 and #3 raised a similar question. At this time the exact mechanism that leads to the decrease in hepatic TG levels following overexpression of miR-33 (as shown in Fig 3) remains to be determined. Gerin et al. (PNAS 2010) and Davalos et al (PNAS 2011) showed that genes involved in fatty acid b-oxidation and triglyceride metabolism (CPT1a, CROT, HADHB, SIRT6 and AMPK1a) are direct targets of miR-33. From these studies it was inferred that miR-33 might function to limit fatty acid utilization in hepatocytes and other cell types, and that sustained elevated levels of miR-33 could lead to fatty liver.

Based on the literature discussed above, we expected to see an increase in hepatic TG contents in mice that overexpressed miR-33, compared to control animals. However, the data was reproducible in 2 independent experiments (n=5/group), and showed a significant decrease in liver TG levels. We can only speculate that the impact of miR-33 on lipid metabolism in vivo is yet to be fully elucidated, both under normal diet conditions and under dietary challenge. Nevertheless, we believe that a lengthy discussion of this topic falls out of the scope of the manuscript and would not add to the story on bile. However, a paragraph has been added on page 9 with the previous discussion.

In response to the reviewer's question, the decrease in Fasn expression in the livers of mice receiving adeno-miR-33 is not due to reduced levels of nSREBP-1 (see Western blot below). Additionally, the putative "target site" for miR-33 or the 3'UTR of SREBP-1 do not confer response to miR-33 in luciferase assays (see data below). Consequently, data do not support the proposal that SREBP-1 is a direct target of miR-33. The reasons behind the decreased expression of Fasn and the reduced hepatic TG levels shown in Fig 3D are not obvious and remain to be established. The new data from SREBP-1 protein levels and on luciferase assays are now included as Fig S3B–D.

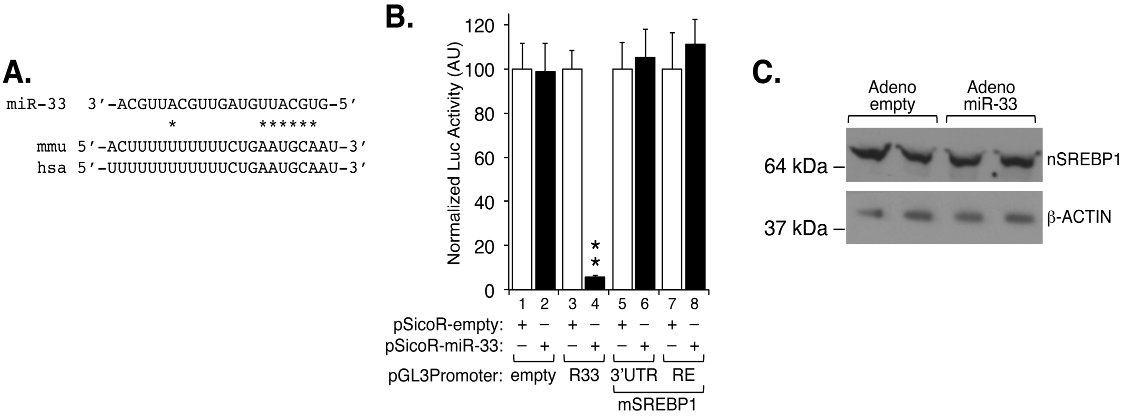

SREBP-1 is not a target of miR-33. ***A.*** *Sequences present in the 3'UTR of human and mouse SREBP1 are complementary to the seed sequence of miR-33.* ***B.*** *The 3'UTR of SREBP-1 (amplified from mouse liver genomic DNA) or oligonucleotides containing the potential miR-33 response element were cloned downstream of a luciferase reporter. Co-transfections of different reporters in HEK293 cells in the presence or absence of a pSicoR plasmid to overexpress miR-33 were performed as described in Methods. The pGL3Promoter-R33 construct contains a sequence with 100% complementarity to miR-33, as described in Figure 2. Data show that neither the 3'UTR nor the putative sequence conferred response to miR-33 overexpression.* ***C.*** *The amounts of nuclear (mature) SREBP-1 were not different in nuclear extracts obtained from the livers of mice transduced with empty and miR-33-encoding adenovirus as in Figure 3. Each lane represent nuclear extracts obtained from 3 pooled livers following the protocol described in* Sheng Z, Otani H, Brown MS, Goldstein JL (1995) Independent regulation of sterol regulatory element-binding proteins 1 and 2 in hamster liver. Proc. Natl. Acad. Sci. USA 92: 935-938.

Major Point 7. The authors state that ABCG5 and ABCG8 are not direct targets of miR-33 given that the target sites in the UTR of these genes do not have "perfect complimentarity". However, there are target sites in the UTR of ABCG5 and ABCG8 that appear to be functional, both according to the luciferase data (Fig S3) and some of the mRNA data (Fig 3F). This may be the result of off-target effects due to the high levels of miR-33 overexpression achieved using adenoviruses, and should be carefully investigated. The authors should also include Western blot data for these genes to support their conclusions that ABCG5/8 are not miR-33 targets.

We agree with the reviewer that a likely explanation for the decrease in hepatic Abcg5 and Abcg8 expression shown in Fig 3F could be an off-target effect due to miR-33 overexpression. As discussed above in Point 5, in these particular experiments where adenoviral transductions are combined with the cholic acid diet the levels of miR-33 are tremendously elevated (approx. 19-fold), compared to controls; however, in other experiments shown in the paper the levels of miR-33 overexpression are 2–4-fold.

Indeed, that is the message that we tried to convey in our original manuscript: on page 9 we wrote "...we could not show repression of these genes [ABCG5 and ABCG8] by adenoviral-mediated miR-33 overexpression either in mouse primary hepatocytes (Fig 2E), nor in human HuH7 or HepG2 cells (Fig S3), nor in human Hep3B cells [where exogenous miR-33 also failed to block the induction of ABCG5 by an LXR agonist (Marquart et al, 2010)]". And later, re the modest decrese in luciferase activity, on the same page: "the physiological relevance of these sequences [in the 3'UTR of ABCG5 and ABCG8] is, however, questionable considering that luciferase assays are performed in the presence of massive overexpression of the miRNA."

At the reviewer's request, we include Western blot data for ABCG5 below and in the new Fig S4, showing that the levels of this transporter do not change in hepatocytes following miR-33 overexpression. The reviewer will notice that the expression of ABCG5 is i) essentially undetectable in control (DMSO) cells, and ii) induced following administration of LXR:RXR agonists (T0901317:9-cis-retinoic acid; 1mM each). Data also show that protein levels for both ABCA1 and ABCG5 are induced by the LXR ligand (as expected), but only the expression of ABCA1 (either basal or LXR-induced) is hindered by miR-33. As discussed in our original manuscript, it was reported that the levels/activity of some canalicular transporters could be, at least under certain conditions, influenced by the relative amounts of each apical transporter in the liver. Hence, Wang et al. [Hepatology

(2003) 38:1489-1499] showed that mice deficient in Abcb11 also have decreased levels of hepatic Abcg5 and Abcg8 when fed a lithogenic diet, through a mechanism that still remains unclear but that seems independent on the levels/activity of LXR. The exact nature of these cross-talk mechanisms between the different biliary transporters remains to be fully elucidated.

The text in the revised manuscript has been amended stating this potential limitation: "(...) We speculate that these latter results are due to non-conserved, imperfect 6-mer sequences (Fig S4) that bind to miR-33 with low affinity. Hence, the decreased expression observed in Fig 3F could be the result of off-target effects due to the supra-physiological levels of miR-33 achieved using adenovirus, thus potentially limiting the interpretation of the results. Intriguingly, mice deficient in ABCB11 show decreased hepatic levels of Abcg5 and Abcg8 when fed a lithogenic diet (Wang et al, 2003); the molecular mechanism of this cross-talk remains unknown, but authors speculate that it is independent on the levels/activity of LXR (Wang et al, 2003). Whether the drop in Abcg5/8 levels in the livers in Fig 3F are due to extreme levels of miR-33, or the result of a yet-unknown signaling pathway that links the expression of different bile transporters will require further investigation."

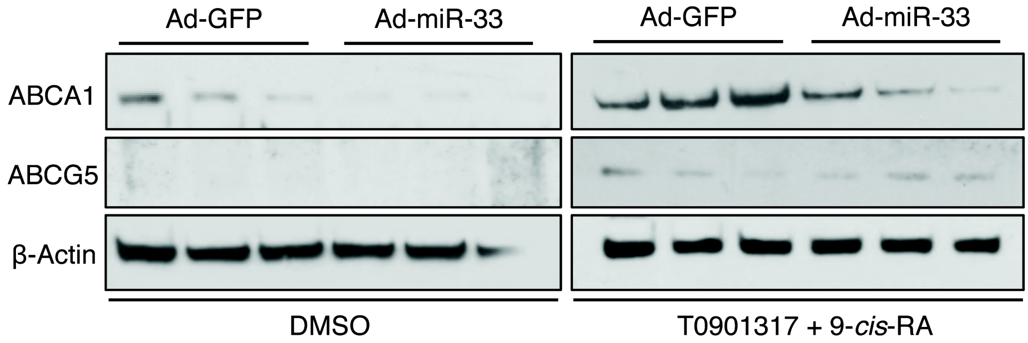

Murine ABCG5 is not a target of miR-33. Mouse primary hepatocytes were isolated and transduced with empty- or miR-33-encoding adenovirus, as described in Methods. Cells were incubated in the presence of DMSO or ligands for LXR:RXR (T0901317:9-cis-retinoic acid; 1μM each) for 24 h. Fifty micrograms of total protein extracts were used to probe the expression of ABCA1 and ABCG5. As expected, both transporters are induced following LXR:RXR activation, but only ABCA1 expression is reduced by miR-33.

Major Point 8. The authors switch from using atorvastatin for the in vivo studies in Fig 5, to simvastatin for the remainder of the in vivo work without providing a rationale. This complicates the interpretation of the data.

At the reviewer's request, we repeated this experiment using simvastatin instead of atorvastatin. As expected the effects of both statins on gene expression were essentially identical. The reviewer will notice that the dose of simvastatin used was 50 mg/Kg/day. It is well documented in the literature that mice respond better to atorvastatin than to simvastatin [reviewed in Zadelaar et al (2007) Mouse models for atherosclerosis and pharmaceutical modifiers. Arterioscler Thromb Vasc Biol 27: 1706-1721]. To satisfy the reviewer's concern, and to strengthen the notion that statins in general repress the expression of miR-33 targets, we now show data from both simvastatin and atorvastatin experiments, and also from HuH7 cells.

© EMBO

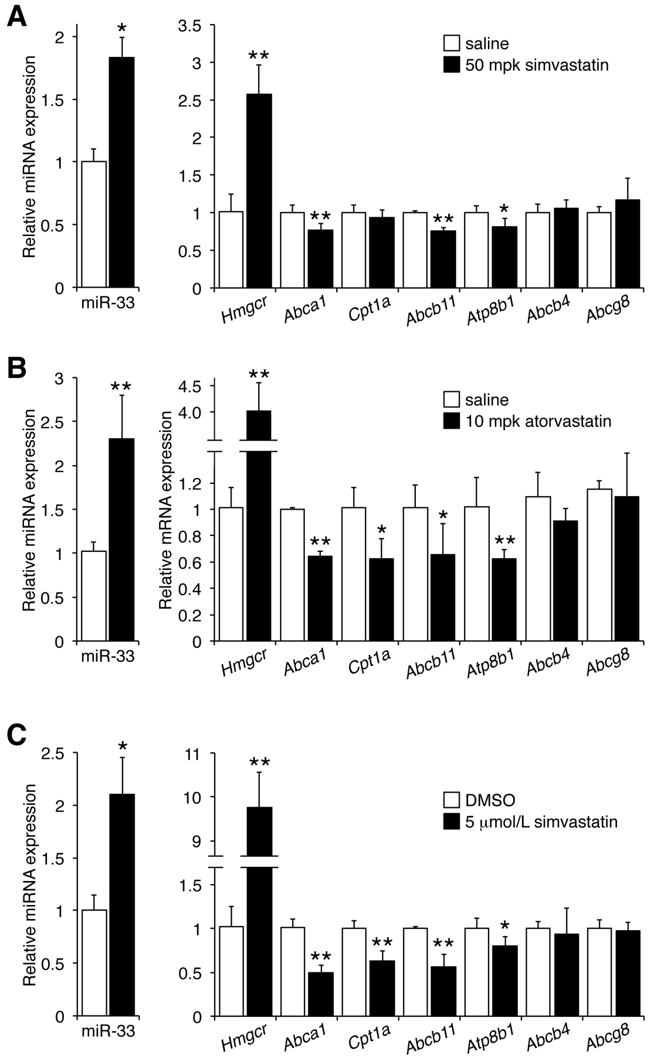

*Statins decrease the mRNA levels of miR-33 targets. C57BL/6 mice (n=5) were gavaged daily for 7 days with 50 mg/Kg (mpk) simvastatin (**A**) or 10 mpk atorvastatin (**B**), and kept on chow diet. Samples were collected following an overnight fasting. **C.** HuH7 hepatoma cells were cultured in DMEM media supplemented with 2% lipoprotein-deficient serum in the presence or absence of 5 μmol/L simvastatin, and total RNA collected after 48 h. Relative expression of specific genes shown as mean ± SD; * $P < 0.05$; ** $P < 0.01$.*

Major Point 9. At several points in the manuscript, the authors assert that effects of miR-33 are specific, as mRNA levels of non-miR-33 targets did not change, however the number of genes examined is quite limited. It is not possible to make such assertions unless more extensive gene expression profiling is performed.

We agree with the reviewer that we used the wrong wording. The text in the manuscript has been changed to state that, within the limited number of transcripts tested, only Abcb11, Atp8b1 and the previously described Abca1 and Cpt1a, responded to miR-33 overexpression or silencing.

Major Point 10. The rationale for the study design presented in Fig 6 & 7 is weak. First, the authors present miR-33 expression data from vehicle, 50 mpk and150 mpk simvastatin, on both a chow and a lithogenic diet (Fig S4). However, 150mpk does not increase miR-33 on a lithogenic diet-yet this was the dose these authors chose to use in combination with anti-miR33 therapy. On page 14, line 12, the authors state "The results from Fig 6 support a mechanism in which statins induce miR-33, which in turn reduces the level of both ABCB11 and ATP8B1, resulting in altered bile

secretion...ultimately leads to liver malfunction". This statement is not supported by the data, where a hepatotoxic dose of simvastatin does not induce miR-33. Moreover, the clinical relevance of these studies is unclear as humans do not consume such a lithogenic diet. Despite the authors' contention that the hepatotoxicity and lethality in mice fed a lithogenic diet concurrent with simvastatin is reminiscent of the recurrent cholestasis found in some patients prescribed statins, this is a highly artificial model that induces tremendous damage to the liver. The mechanisms by which anti-miR-33 exert its positive effects under these conditions are not clear.

We apologize for the confusion re the effect of simvastatin and lithogenic diet on miR-33 expression. Data in Fig. S5A show that both 50 and 150 mg/Kg/day for 2 days (days -2 and -1, on chow) elevate the levels of hepatic miR-33, compared to saline. It was after these 2 days on chow plus/minus the statin that we switched the mice to the lithogenic diet. Data in Fig. S5B show the levels of hepatic miR-33 at the completion of the experiment (day 7 on the diet). We do not know how the levels of miR-33 evolve over time (i.e. each day) in the mice receiving 150 mpk simvastatin, but what we do know is that those are the mice that start with highest levels of miR-33 at day 0. The reviewer will notice that: i) the livers of mice dosed with 150 mpk simvastatin are very enlarged and steatotic (Fig. 6B–D); and ii) there is a great variability in the expression levels of most hepatic transcripts analyzed in mice receiving 150 mpk simvastatin, with several mRNA plummeting specially in those mice that had to be sacrificed before day 7 (data not shown in Fig 6, but they are essentially identical to those shown in Fig 7G for mice receiving scrambled oligos). We can only speculate that the expression of miR-33 declines over time in mice receiving the highest dose of the statin, not because the statin does not induce Srebp-2/miR-33 expression, but because of the toxic effects of the drug in combination with the diet, and that both factors combined compromise the normal function of the hepatocyte. Consequently, we disagree with the reviewer when (s)he states that "a hepatotoxic dose of simvastatin does not induce miR-33".

Indeed, the involvement of miR-33 in (at least part of) the statin- and diet-mediated hepatotoxicity is conclusively revealed by the rescue effect of the antisense oligos (Fig 7). We proposed that the effect of miR-33 on bile secretion through Abcb11 and Atpt8b1 helps explain the effect of the antisense treatment, but we also stated that "whether other miR-33 targets besides Abcb11 and Atp8b1 or other, bile-independent pathways are also necessary for this effect remains to be established". But we agree with the reviewer that the exact positive actions of anti-miR-33 are not completely clear. Consequently, we modified that paragraph to: "the exact hepatoprotective mechanism of anti-miR-33 on statin- and diet-induced toxicity is yet to be determined. We speculate that increased bile flow due to de-repression of miR-33 targets such as Abcb11 and/or Atp8b1 might contribute to the protective effect, but whether other bile-independent pathways are also necessary for hepatoprotection remains to be established".

We also agree with the reviewer that humans do not consume a lithogenic diet and, consequently, our model is highly artificial. Consequently, the description of these experiments as reminiscent of statin-induced cholestasis in patients has been toned down in the discussion. The new text in that section now reads (new text underlined): "Since SREBP-2/miR-33 are transcriptionally induced following treatment with statins (Marquart et al, 2010; Najafi-Shoushtari et al, 2010; Rayner et al, 2010), we hypothesized that miR-33 might account for some of the (side) effects of these drugs. Reports show that certain patients develop cholestasis following prescription of statins (Batey & Harvey, 2002; de Castro et al, 2006; Merli et al, 2010; Rahier et al, 2008; Ridruejo & Mando, 2002; Torres et al, 2002). While the molecular events in the livers of these patients are unknown, this clinical setting is consistent with our model (Fig 8) in which statin-induced miR-33 represses both ABCB11 and ATP8B1, thus decreasing bile secretion and eventually leading to intrahepatic cholestasis. We recognize, however, that our experimental model is extreme due to the relatively high dose of the statin used and the fact that patients do not normally consume lithogenic diets (i.e., a high fat, high cholesterol, high bile acid diet). It is intriguing to speculate, however, that these patients might carry specific polymorphisms in ABCB11 or ATP8B1 that would make them more susceptible to statin-induced miR-33 effects. In general, most physicians are cautious to prescribe statins to patients with underlying liver disease, and the effect of these drugs on patients with primary biliary

cirrhosis is controversial (Abu Rajab & Kaplan, 2010; Stanca et al, 2008; Stojakovic et al, 2010; Stojakovic et al, 2007). Nevertheless, our data clearly demonstrate a dose-dependent effect of simvastatin on diet-induced hepatotoxicity and cholestasis in mice. It is important to stress the fact that mice tolerate 300 mpk statins with a concomitant high fat, high cholesterol diet for months (Zadelaar et al, 2007), but to our knowledge no previous studies addressed the interaction between statins and cholate feeding. Our data offer conclusive evidence that, at least in mice, statins potentiate diet-induced cholestasis. From a mechanistic perspective, our data show that the phenotype is largely rescued by anti-miR-33 oligonucleotides."

We hope the new text is acceptable for the reviewer.

Major Point 11. On p.14 of the results, the authors state that it is unclear whether the decrease in Abcb11 in the livers of statin treated mice is FXR-independent, since the expression of other FXR target genes was either reduced (Shp) or unchanged (Abcb4). By saying this, the authors appear to be challenging their own hypothesis that these changes are occurring through statin-induced upregulation of miR-33. Was FXR protein level measured? The protein levels of the genes shown in figure 6G should be presented.

We acknowledge that our choice of words was not the best there. Our statement originated from the fact that Abcb11, Abcb4 and Shp are all "classic" FXR targets that are typically regulated coordinately in response to changes in activation of this nuclear receptor. Since we found the levels of Shp to be decreased in those livers, one might think that there was less active FXR (or less FXR protein) and, consequently, the drop in Abcb11 levels could be due to decreased FXR activity on the Abcb11 promoter (and perhaps independent on miR-33). However, the other FXR target, Abcb4, shows no change in mRNA levels between groups, suggesting that levels of active FXR do not change. The fact that these two FXR targets (Shp and Abcb4) show different patterns of expression in response to the treatment limits the interpretation of the data for Abcb11 in this particular experiment. However, we show in the new Fig. 5 that both simvastatin and atorvastatin decrease the expression of Abcb11 without changing the expression of Abcb4. Taken together, the data strongly suggest that the decrease in Abcb11 expression in these mice is driven by miR-33, and not by changes in active FXR levels.

Upon revision, we think our original sentence was clearly confusing, and we decided to remove it from the new version of our manuscript. At the reviewer's request, we measured the levels of hepatic FXR in mice gavaged with saline or 50 mpk simvastatin (see Western blot below). Data show that there were no changes in the levels of this protein between groups. Since we removed the original comment form the manuscript, we do not include this western blot data in the new manuscript.

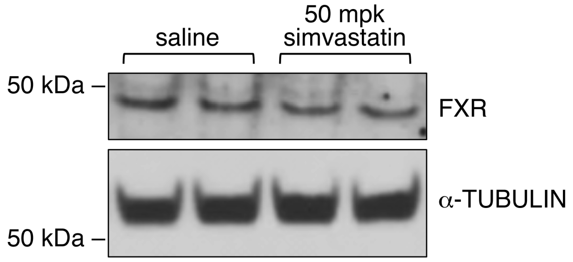

The levels of FXR protein are not changed in mice treated with simvastatin. Mice were treated as described in Figure 5. The abundance of FXR was measured in 50 μg total liver extracts from individual mice, using a specific anti-FXR antibody (SCBT #13551; 1:200 dilution), as described in Methods.

Major Point 12. In Fig. 7G, it is unclear how anti-miR-33 treatment causes such a dramatic rescue of Abcb4 levels when this gene is not a target of miR-33. Again, the variability of Abcg5 and G8 measurements are concerning and the mechanism by which anti-miR-33 treatment affects these genes if they are not direct targets is unclear. The figure legend should clearly state the number of mice in each group (moribund, alive and anti-miR-33).

We revised the text of the legend to state the number of mice on each group: 5 moribund, 5 alive and 10 anti-miR-33.

The reviewer is right when s/he points to the fact that the tremendous variation in mRNA expression between animals limits the interpretation of data in Fig 7G. However, the variability in other parameters measured in these mice (body weight, liver to body mass ratio, hepatic lipid contents) was relatively tight (see rest of Fig 7 and Fig S6). We do not have the answer to why mRNA expression is so variable for bile transporters, but it is certainly surprising. We can only speculate, as we did in our original manuscript, that the expression of bile-related genes (including not only miR-33 targets, but also Abcb4 and Abcg5/8) is particularly critical to support the viability of the mice under conditions of dietary challenge and statin-induced toxicity. That would explain that mice that can maintain the expression of those bile genes above a certain threshold are able to survive the treatment, while those that fail to sustain the expression of bile transporters succumb to the treatment. Expression data in Fig 7G and S6D certainly supports that hypothesis. The main text includes now a brief discussion with this argument.

Minor Point 1. Fig. 1E is not included in the figure legend

We apologize for that oversight. The text in the legend has been corrected.

Minor Point 2. Legend for Figure 4 should be modified to indicate the time point at which samples were taken for c-e

The text in the legend has been revised to include this information.

2nd Editorial Decision 10 May 2012

Thank you for the submission of your revised manuscript to EMBO Molecular Medicine. We have now received the enclosed reports from the referees that were asked to re-assess it. As you will see the reviewers are now supportive and I am pleased to inform you that we will be able to accept your manuscript pending the following final amendments:

- Referee #1 still has a few minor concerns that I would like you to address.

- In addition, please provide each individual figures in the way you would like them to appear in the manuscript. For example, figure 1 might fit better in a portrait format where the different graphs will be bigger and more readable. Similar comment applies to most of the figures. We rarely use a landscape format for figures. Keep in mind that figures might be reduced or blown up to fit the copy editing of the article. Make sure that all labels remain readable even if a figure is reduced, and resolution remains optimal even if the figure is blown up.

Please submit your revised manuscript within two weeks.

I look forward to reading a new revised version of your manuscript as soon as possible.

Yours sincerely,

Editor
EMBO Molecular Medicine

***** Reviewer's comments *****

Referee #1:

The authors put a lot of effort into answering all questions of the reviewers to fulfil their suggestions

and comments. Some important additional data such as the expression of specific hepatic canalicular transporters after simvastatin treatment or the protein expression of SREBP-1 in liver were added which support the underlying hypothesis. In general the paper is well-structured, and I have only few minor additional points.

- Figure 1E is still not included in the MS text. The authors write that data in Fig 1D show that the secretion rates of total bile, bile acids and phosphatidylcholine were significantly increased in mice injected with anti-miR-33 oligonucleotides compared to control animals. However the secretion rates of bile acids, Pc and chol is depicted in Fig 1E. Please correct that.
- In Figure 4A it can not be seen what the white and black bars mean. It is written in the Figure legend, but it would be easier for the readers if this is stated next to the figure as the authors did for other figures such as Fig 1A or 3A. Please correct that also for Fig S2.

Referee #3:

The authors have responded adequately to the critiques

2nd Revision - Authors' Response 16 May 2012

Responses to Reviewer #1.

Point 1. Figure 1E is still not included in the MS text. The authors write that data in Fig 1D show that the secretion rates of total bile, bile acids and phosphatidylcholine were significantly increased in mice injected with anti-miR-33 oligonucleotides compared to control animals. However the secretion rates of bile acids, Pc and chol is depicted in Fig 1E. Please correct that.

The MS text has been revised, and now reads "Data in Figs 1D, E show that the secretion rates of total bile, bile acids, and phosphatidylcholine were significantly increased in …".

Point 2. In Figure 4A it can not be seen what the white and black bars mean. It is written in the Figure legend, but it would be easier for the readers if this is stated next to the figure as the authors did for other figures such as Fig 1A or 3A. Please correct that also for Fig S2.

Figures 4A and S2 have been revised.
