# Supplementary material for: miR-33 controls the expression of biliary transporters, and mediates statin- and diet-induced hepatotoxicity
Source: EMBO Mol Med. 2012 Jul 5;4(9):882–95. doi: 10.1002/emmm.201201228 (PMC3491822; doi:10.1002/emmm.201201228)
Supplement: Supplementary file 2 [file emmm0004-0882-SD2.pdf]

**SUPPORTING INFORMATION related to**

# miR-33 controls the expression of biliary transporters, and mediates statin- and diet-induced hepatotoxicity

Ryan M. Allen, Tyler J. Marquart, Carolyn J. Albert, Frederick J. Suchy, David Q.-H. Wang, Meenakshisundaram Ananthanarayanan, David A. Ford, and Ángel Baldán

## Table of contents

Supporting Figure Legends ………………………………………………………….. 2
Supporting Figures ………………………………………………………….. 5
Supporting References ………………………………………………………….. 11

## SUPPORTING FIGURE LEGENDS

**Figure S1. Expression profile of selected hepatic genes following treatment with anti-miR-33 oligonucleotides.** C57BL/6 mice (n=5) were injected with saline, or scrambled or anti-miR-33 oligonucleotides (5 mpk i.v., for 3 consecutive days), fed chow for 7 days, and then fasted overnight before sacrifice. Relative hepatic mRNA expression of genes involved in bile metabolism and of two previously described miR-33 targets were determined by real time qPCR. Data are shown as mean ± SD; ** $P < 0.01$

**Figure S2. Bile acid secretion and synthesis are decreased in cells overexpressing miR-33.** HuH7 cells were seeded in 24-well plates and transduced with empty (open bars) or miR-33 (closed bars) adenovirus. The next morning, cells were pulsed in media supplemented with 0.2% BSA and cyclodextran:cholesterol spiked with [$^{14}$C]-cholesterol (prepared as described in Christian *et al.*, 1997). The final specific activity in the media was 2 µCi/mL. After 6 h, cells were washed in PBS and chased in fresh media supplemented with 0.2% BSA for 24 or 48 h. Chloroform- and water-soluble radiolabeled sterols (cholesterol and bile acids, respectively) were separated as described in Methods for the RCT experiment. Figure shows data from 8 replicas per condition. ** $P < 0.01$ miR-33 *vs*. empty; ¶ 48 h *vs*. 24 h.

**Figure S3. Hepatic expression of selected genes involved in lipid metabolism in mice overexpressing miR-33**.

**A.** C57BL/6 mice (n=5) were transduced with empty or miR-33 encoding adenovirus vectors and then switched to a lithogenic diet, as described in *Fig. 3*. The relative abundance of selected genes was determined by real time qPCR.

**B.** Potential miR-33 response element in the 3'UTR of mouse and human *SREBP-1*. Asterisks denote complementarity to miR-33.

**C.** The 3'UTR of mouse *Srebp-1*, or the potential murine miR-33 responsive element shown in panel B were cloned downstream of a luciferase reporter. The effect of miR-33 overexpression on Luc activity was assayed as described in *Fig. 2*. Negative control (empty luc vector) and a positive control (reporter containing a 100% match to miR-33) were also included in these experiments.

**D.** Nuclear extracts were prepared from the livers of the same mice (as described in Sheng et al, 1995). Pieces from 2 livers were pooled to generate each nuclear extract sample. Fifty μg of total protein were separated by SDS-PAGE and probed with antibodies against the mature SREBP1, and β-actin.

**E.** C57BL/6 mice (n=5) were transduced with empty or miR-33 encoding adenovirus vectors and kept on a standard chow diet. The relative abundance of selected genes was determined by real time qPCR. Data are shown as mean ± SD; nd, not detected; * $P < 0.05$; ** $P < 0.01$; nd, not detected.

**Figure S4. Analysis of mouse and human *ABCG5* and *ABCG8* as potential direct targets of miR-33.**

**A.** Human HepG2 and Huh7 hepatoma cells were transduced with empty or miR-33- encoding adenovirus, and the expression of *ABCG5* and *ABCG8* was measured 48 h later by real time qPCR.

**B.** The 3'UTR of mouse and human ABCG5 and ABCG8 were amplified from genomic DNA and cloned downstream of a luciferase reporter. The effect of miR-33 overexpression on Luc activity was assayed as described in *Fig. 2*. Negative control (empty luc vector) and a positive control (reporter containing a 100% match to miR-33) were also included in these experiments.

**C.** Human Huh7 hepatoma cells were transduced with empty or miR-33-encoding adenovirus, and then incubated in the presence or absence of agonists for LXR:RXR [T0901317:9-*cis*-retinoic acid; 1μmol/L each] for 48 h. The protein levels of ABCA1 and ABCG5 were measured by Western blot using 50 μg of total protein extract.

**D.** Analysis of the 3'UTR of human and mouse *ABCG5* and *ABCG8*. Sequences in red and underlined are partially complementary to the seed sequence of miR-33. Sequences in italics and underlined are polyA signals. Data are shown as mean ± SD; * $P < 0.05$; ** $P < 0.01$.

**Figure S5. Simvastatin and lithogenic diet induce liver toxicity.**

**A.** C57BL/6 mice (n=4) were gavaged with simvastatin and kept on chow for a week. The expression of miR-33 was normalized to that of U6 snRNA

**B.** C57BL/6 mice (n=4) were gavaged with simvastatin and fed a lithogenic diet as described in *Fig. 6*. The expression of miR-33 was normalized to that of U6 snRNA.

**C.** Daily body weight change (n=6).

**D.** Daily average food consumption (n=6).

**E.** Lipidomics analysis of hepatic contents (n=3) was performed by ESI-M. Individual molecular species were quantified by comparisons to the internal standards as described in Methods.

**F.** Lipidomics analysis of plasma (n=3).

Data are shown as mean ± SD.

**Figure S6. Silencing miR-33 rescues the liver damage induced by simvastatin and lithogenic diet.** C57BL/6 mice (n=12) were injected with scrambled or anti-miR-33 oligonucleotides, and subsequently dosed with simvastatin and fed a lithogenic diet, as described in *Fig. 7*.

**A.** Relative expression of miR-33, normalized to U6 snRNA (n=4).

**B.** Lipidomics analysis of hepatic contents (n=3) was performed by ESI-MS. Individual molecular species were quantified by comparisons to the internal standards as described in Methods.

**C.** Lipidomics analysis of plasma (n=3).

**D.** Relative expression of detoxifying genes in the same mice (n=5 for each group of scrambled; n=10 for the anti-miR-33 group). The expression of *Oatp4* and *Car* was significantly higher in the antisense group, compared to both scrambled groups. The expression of most transcripts, with the exception of *Cyp3a11* and *Mrp4,* was significantly decreased in the moribund group.

Data are shown as mean ± SD.

**SUPPORTING FIGURES**

**Figure S1**

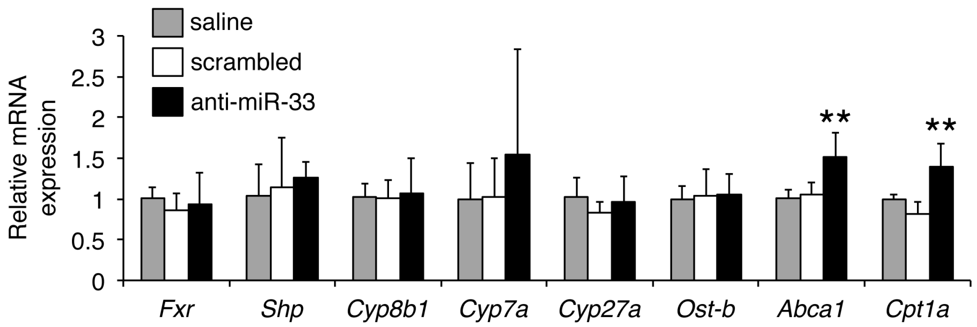

## Figure S2

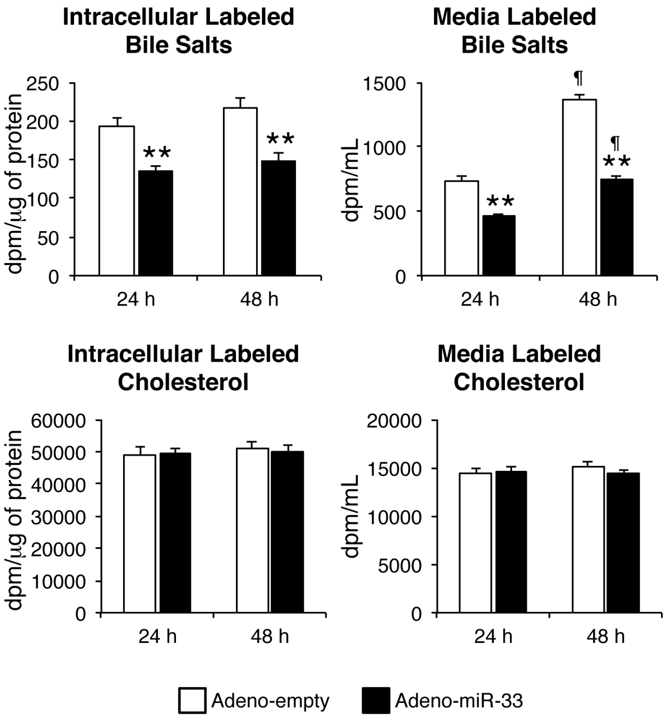
Intracellular Labeled Bile Salts
dpm/µg of protein
250
200
150
100
50
0
**
**
24 h
48 h
Media Labeled Bile Salts
dpm/mL
1500
1000
500
0
¶
¶
**
**
24 h
48 h
Intracellular Labeled Cholesterol
dpm/µg of protein
60000
50000
40000
30000
20000
10000
0
24 h
48 h
Media Labeled Cholesterol
dpm/mL
20000
15000
10000
5000
0
24 h
48 h
Adeno-empty
Adeno-miR-33

# Figure S3

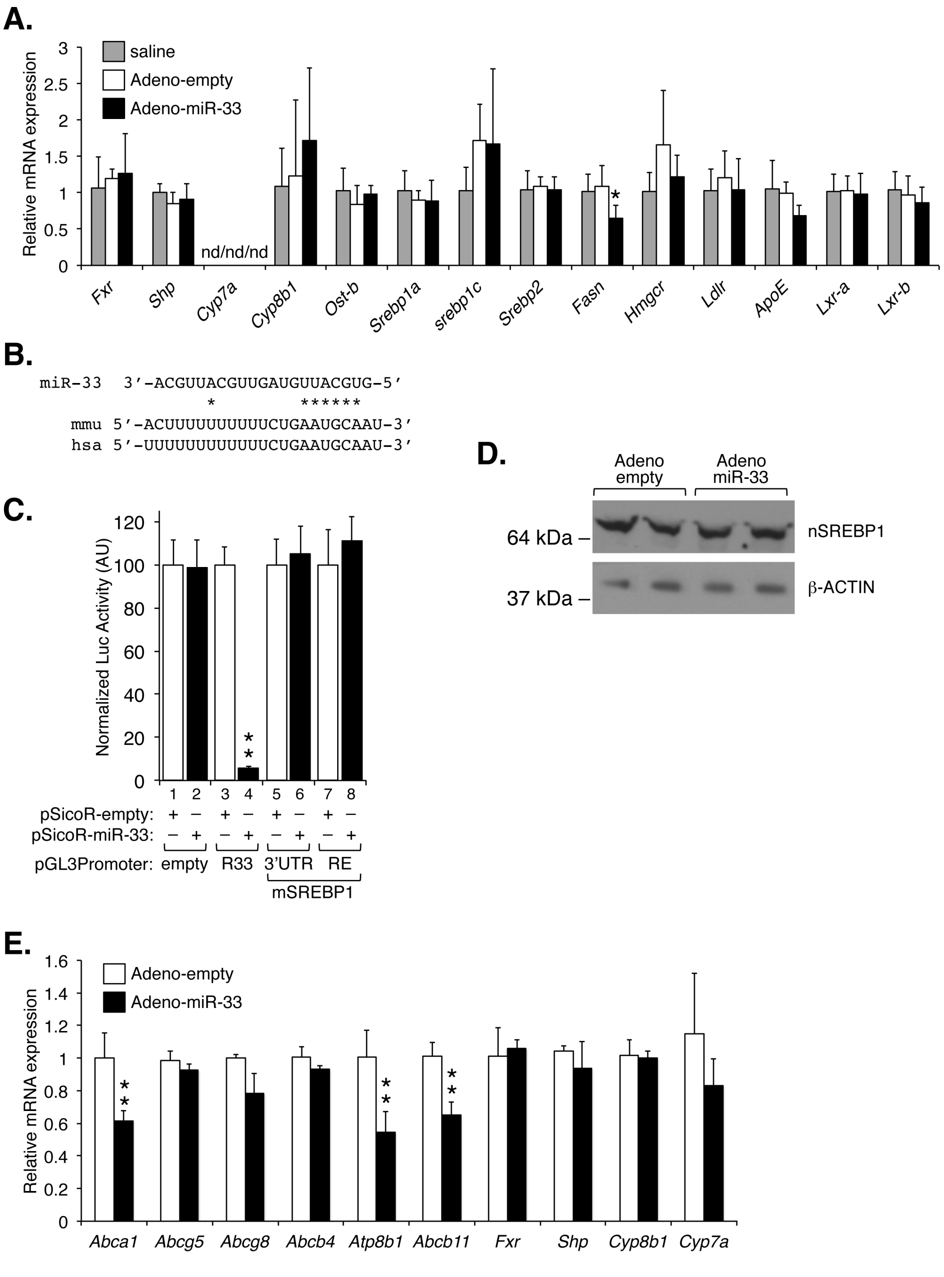
A.
saline
Adeno-empty
Adeno-miR-33
Relative mRNA expression
3
2.5
2
1.5
1
0.5
0
nd/nd/nd
*
Fxr
Shp
Cyp7a
Cyp8b1
Ost-b
Srebp1a
srebp1c
Srebp2
Fasn
Hmgcr
Ldlr
ApoE
Lxr-a
Lxr-b
B.
miR-33 3'-ACGUUACGUUGAUGUUACGUG-5'
* ******
mmu 5'-ACUUUUUUUUUUUCUGAAUGCAAU-3'
hsa 5'-UUUUUUUUUUUUUCUGAAUGCAAU-3'
C.
Normalized Luc Activity (AU)
120
100
80
60
40
20
0
**
1 2 3 4 5 6 7 8
pSicoR-empty: + − + − + − + −
pSicoR-miR-33: − + − + − + − +
pGL3Promoter: empty R33 3'UTR RE
mSREBP1
D.
Adeno empty
Adeno miR-33
64 kDa –
37 kDa –
nSREBP1
β-ACTIN
E.
Adeno-empty
Adeno-miR-33
Relative mRNA expression
1.6
1.4
1.2
1
0.8
0.6
0.4
0.2
0
**
**
**
Abca1
Abcg5
Abcg8
Abcb4
Atp8b1
Abcb11
Fxr
Shp
Cyp8b1
Cyp7a

S

# Figure S4

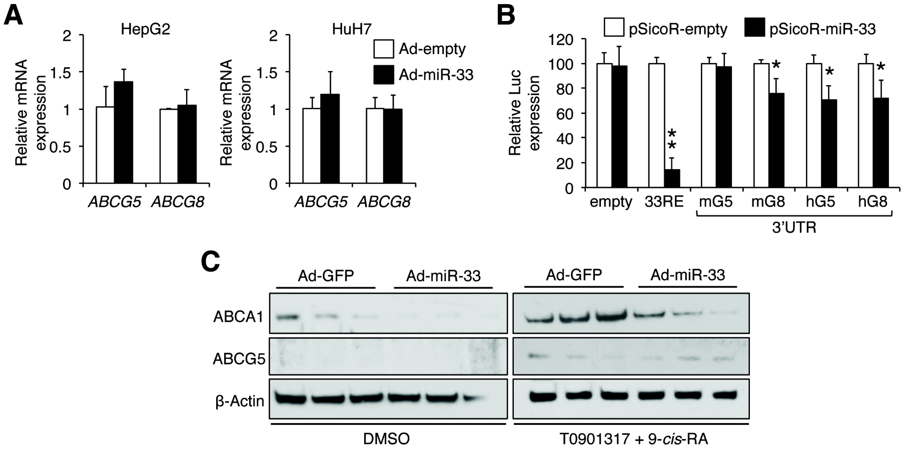


**D**

```
miR-33: 3'-ACGUUACGUUGAUGUUACGUG-5'
                          |||||||
seed target: 5'-NNNNNNNNNNNNNCAAUGCAN-3'
```

**hsa *ABCG5* 3'UTR:**

aagccauggcugggaaAAUGgAagugaagcugccgacugugcaugacugcucugaacgucugaaaugagagugccauguauuucuuucuugacaggacaucucaagucuuuuaaccau
uaagacuccauuugugccucuuggauccaagcaggccuugAAUGCAauggaagugguuuauagucccuugcucuuacaacuugcagggacaugugguuauuuggaaauugugacugag
cggacccaagAAUGuAaauaauauucauaaaccuaugggagacucgugugacuauuuuuuuuccuuguucuaggcacagaaaaaaaauaggucagcuuaaaaauauguuuacauuggau
aaaggauuaggcaaa*aauaaa*auguuucaaggauuccugaccauaagugacagagaaagagaguuguggguuuagaugaagcaagguuaucaugcagaauugggguaagaaugcuucug
uuccuggaagacccagaguuaAAUGCAgauguccacacgaggggucggaguuaccugaucacaucgagagagugcugggcagauggauggugagcaccacugcuacagagcacccagu
gauuuuacugagg*auuaaaauaaa*aaaccguaggaaugggcucaacaguga

**mmu *Abcg5* 3'UTR:**

guuaagaugacaggcaggaaaggguuaaugggcaggcacgcccacuguggagcacagagaaguacugucuacaaccaucaggauuccaucugcgacccuugugucugacccuuguguc
uauccggagccccaagggcaacgagaacucacagcccucugcuauuccagcuuguggggcaaugugguguuggacauugugacugaacugguccaauAAUGuA*aauaauaauaau*uc
auaaaccuacaggacauua

**hsa *ABCG8* 3'UTR:**

caagacuggugauucacgccagacgucugcccgcugguggggaccugagcagacccuucaacugcacucccuccucaggagccccuuccuggggacagugaggacaaugacccuaca
gaugcucagcuacauccggcccagggugcugcaguggcacagaccagccacaggauggcaguag*aauaaa*gacagucgaaagggauuucugcucacuggcaggagacugcgaugacug
ggagaaaaccugcacucgguggcaccuacaacguugcuaauuuauuuccuuuugauaugcauuuauauaggcaacucgauauaggaugggagcaaacuaggAAUGaAuuggguagcua
gacugugcaggaauuguuggaaccuggagggaacaauaacaguagcuagcagauuuggcuucaucuuccaggggccccacacuccguggugagccaccaucaauacagaaagugaccu
aagauguaccagcaagaugccaucccuucuuuuugugugggggucaugggcuccaaaagccaacgugaaca*auuaaa*AAUGuAuugagcaucua

**mmu *Abcg8* 3'UTR:**

uacucagccuugcucucacuggcgggacccuuuucccggggcuggccaccccaggaggagccggacuggggacaaggcucacacagaucucucaggcagcagccaccucuuagugcug
caguggcacaggucagccacaggauggcaguag*aauaaa*gacaguugagagguguuucugcucccaggcccaggcuugugaugggagagagagaaaccagguacguugcucaugcauu
uuauaucuuua*aauaaa*caacccaguauggaaugggaaccaauuauauaugaauugaguagcuaggcuaugcagaaauuucuggaauccugagaggauaguggguuuauagcaaagugu
uuaacuuucucuucuaccauucucacacuguuaagccacucccaauacaaagggcgaccuaaaacaaacuagcaaaauguuuuucgcuuaucucugcguggauucauggacuccaacc
cccaaaguuggacagcugagaguuaauugagcauuuaaucaacggguacgguauaaaauaugaugugugggcuauugucauuuauucuuggagcaacccacugaugugagcagcauccu
guuauuucggugaggacacgguggcaaagacaaauccaguugucucagcuuacaaaguguuuugugauggaaucaaaaagacccuugggcguuacagagcaaaacgccaggcccgaga
ugccucuagaaacaguuugcagagucugugugagcucagccaggccucagaguugcaucccccuagccagggcucaggaucggcuuucacaguggccagagaugcgcugcuugcugca
cauccagauugcgugcuucaugccugugucaaggaaccccaccccaccccuggccccccaccccgggcccccgacccaaagcauggagcugggaggggcuugcucccagaggaaaac
ucgucuggAAUGaAaagaaccagcggggagaguguuugaggccaggagagguguucuccacugguucuccagguccugauugguccaugacgucagugaaugcugaugagguaggcaa
aaucccaggccugcccagauccguggacucagcauccuguguucagaccccagugugagcagcagcuccacagcauccacuggccucagggaguccacacagcccuguugccaccauc
cauugcauagaugucacacaagagccacagggcccucaugcagagcagagucaaaagcacccuccgauugcuucuuucaguuuggaagagcuagcucuucagugcccgcuggcuuuau
aggguagcuuuuaauuuggauuuaucugggggcauuucucaguuaaauccuggcuggguucagaauauccaggAAUGaAauugguuuuucccuagugugucccaucaggcggcacacgg
uuggcucccaguugcugcgugcuguugacucugauugccugacagaaggcacgucugcuggaccccuccacguuccucgguucccucuuuucauaauu*aauaaa*gcuuuuggauacca

# Figure S5

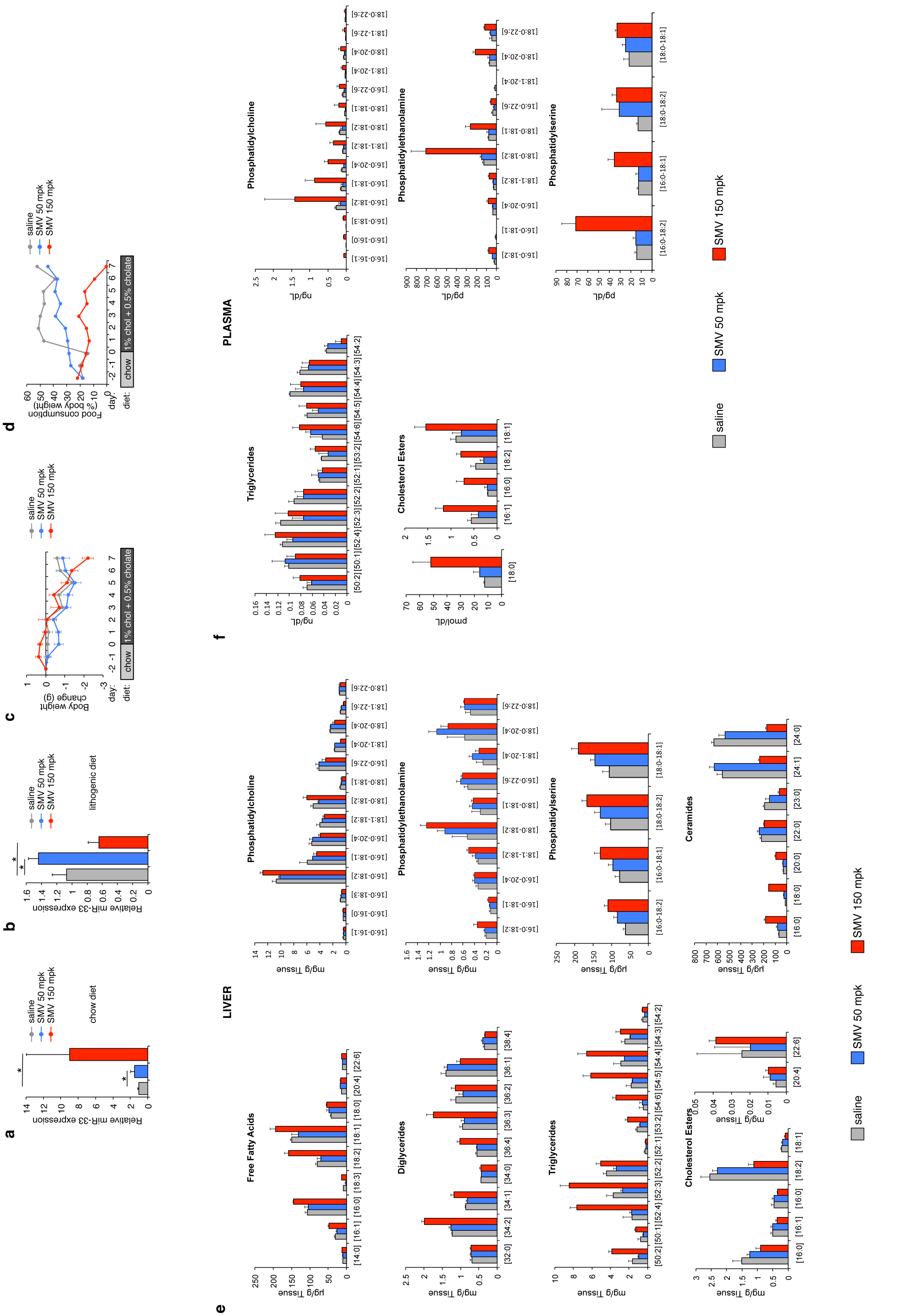
a
b
c
d
e
f
Relative miR-33 expression
saline
SMV 50 mpk
SMV 150 mpk
chow diet
lithogenic diet
Body weight change (g)
Food consumption (% body weight)
day:
diet:
chow
1% chol + 0.5% cholate
LIVER
PLASMA
Free Fatty Acids
Diglycerides
Triglycerides
Cholesterol Esters
Phosphatidylcholine
Phosphatidylethanolamine
Phosphatidylserine
Ceramides
µg/g Tissue
mg/g Tissue
ng/dL
pmol/dL
pg/dL

# Figure S6

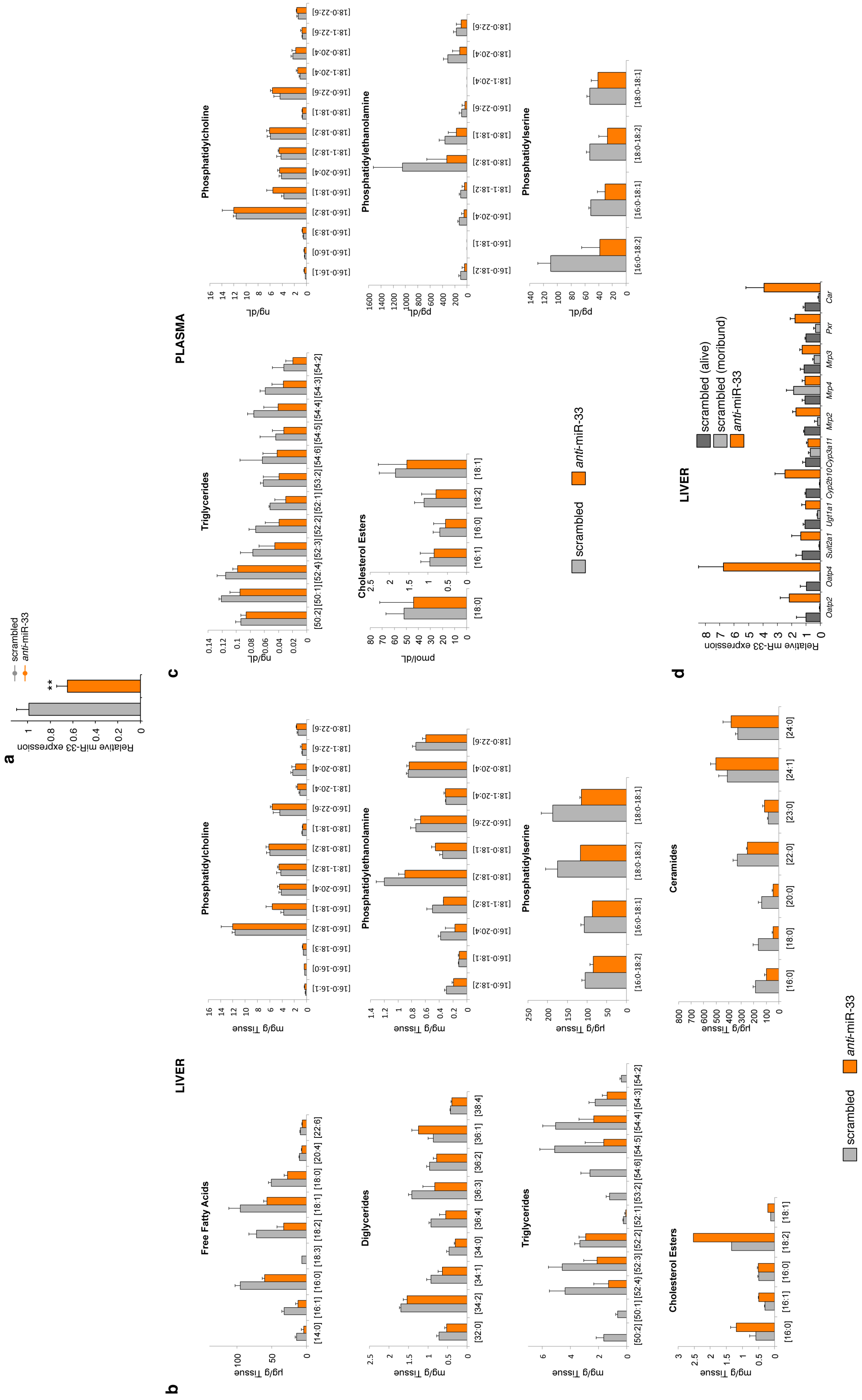
a
Relative miR-33 expression
scrambled
anti-miR-33
b
LIVER
Free Fatty Acids
Diglycerides
Triglycerides
Cholesterol Esters
Phosphatidylcholine
Phosphatidylethanolamine
Phosphatidylserine
Ceramides
scrambled
anti-miR-33
c
PLASMA
Triglycerides
Cholesterol Esters
Phosphatidylcholine
Phosphatidylethanolamine
Phosphatidylserine
scrambled
anti-miR-33
d
LIVER
scrambled (alive)
scrambled (moribund)
anti-miR-33
Relative mR-33 expression

**SUPPORTING REFERENCES**

Christian AE, Haynes MP, Phillips MC, Rothblat GH (1997) Use of cyclodextrins for manipulating cellular cholesterol content. J Lipid Res 38:2264-2272.

Sheng Z, Otani H, Brown MS, Goldstein JL (1995) Independent regulation of sterol regulatory element-binding proteins 1 and 2 in hamster liver. Proc. Natl. Acad. Sci. USA 92: 935-938.
